# Glutamine metabolism is essential for coronavirus replication in host cells and in mice

**DOI:** 10.1016/j.jbc.2024.108063

**Published:** 2024-12-09

**Authors:** Kai Su Greene, Annette Choi, Nianhui Yang, Matthew Chen, Ruizhi Li, Yijian Qiu, Shahrzad Ezzatpour, Katherine S. Rojas, Jonathan Shen, Kristin F. Wilson, William P. Katt, Hector C. Aguilar, Michael J. Lukey, Gary R. Whittaker, Richard A. Cerione

**Affiliations:** 1 Department of Molecular Medicine, Cornell University, Ithaca, New York, USA; 2 Department of Microbiology and Immunology, Cornell University, Ithaca, New York, USA; 3 Cancer Center, Cold Spring Harbor Laboratory, Cold Spring Harbor, New York, USA; 4 Public & Ecosystem Health, Cornell University, Ithaca, New York, USA; 5 Department of Chemistry and Chemical Biology, Cornell University, Ithaca, New York, USA

**Keywords:** glutamine metabolism, SARS-CoV-2, HCoV-OC43, HCoV-229E, GLS, GLS2, pan-glutaminase inhibitor SU1, GLS inhibitor UP4

## Abstract

Understanding the fundamental biochemical and metabolic requirements for the replication of coronaviruses within infected cells is of notable interest for the development of broad-based therapeutic strategies, given the likelihood of the emergence of new pandemic-potential virus species, as well as future variants of SARS-CoV-2. Here we demonstrate members of the glutaminase family of enzymes (GLS and GLS2), which catalyze the hydrolysis of glutamine to glutamate (*i.e.*, the first step in glutamine metabolism), play key roles in coronavirus replication in host cells. Using a range of human seasonal and zoonotic coronaviruses, we show three examples where GLS expression increases during coronavirus infection of host cells, and another where GLS2 is upregulated. The viruses hijack the metabolic machinery responsible for glutamine metabolism to generate the building blocks for biosynthetic processes and satisfy the bioenergetic requirements demanded by the “glutamine addiction” of virus-infected cells. We demonstrate that genetic silencing of glutaminase enzymes reduces coronavirus infection and that newer members of two classes of allosteric inhibitors targeting these enzymes, designated as SU1, a pan-GLS/GLS2 inhibitor, and UP4, a specific GLS inhibitor, block viral replication in epithelial cells. Moreover, treatment of SARS-CoV-2 infected K18-human ACE2 transgenic mice with SU1 resulted in their complete survival compared to untreated control animals, which succumbed within 10 days post-infection. Overall, these findings highlight the importance of glutamine metabolism for coronavirus replication in human cells and mice and show that glutaminase inhibitors can block coronavirus infection and thereby may represent a novel class of broad-based anti-viral drug candidates.

Coronaviruses are a group of enveloped viruses that contain a single positive RNA strand and “corona”-like spike proteins extending from their envelopes. Seven types of coronaviruses have been reported to infect humans (1, 2). In 2019, the severe acute respiratory syndrome coronavirus 2 (SARS-CoV-2) triggered a worldwide pandemic, with multiple viral variants having emerged since that time. Human coronaviruses OC43 (HCoV-OC43) and 229E (HCoV-229E) are far less lethal and typically only give rise to common cold symptoms. Each coronavirus family member has a distinct spike protein and consequently binds to different receptors to enter cells. SARS-CoV-2 binds to the angiotensin-converting enzyme 2 (ACE2) receptor (3, 4, 5), HCoV-OC43 engages the 9-*O*-acetylated sialic acid (9-*O*-Ac-Sia) receptor, and HCoV229E interacts preferentially with human aminopeptidase N (hAPN) (4, 5, 6, 7, 8, 9, 10, 11). SARS-CoV-2 and HCoV-OC43 are members of the coronavirus beta sub-group family, while HCoV-229E is a member of the alpha sub-group. Since the onset of the pandemic, therapeutic antibodies and vaccines targeting SARS-CoV-2 have been developed, although each has its limitations. Thus, there continues to be a pressing need to identify new therapeutic strategies that will provide broad protection against new virus strains and mutants that are likely to emerge.

Viruses have been suggested to reprogram the metabolism of host cells to support their bioenergetic requirements for replication, raising the possibility that targeting metabolic activities essential for viral infections might offer potential therapeutic strategies. For example, there have been reports suggesting that some viruses are dependent upon glutamine metabolism for their replication and the generation of new viral particles including human cytomegalovirus (HCMV), Kaposi-sarcoma-associated herpesvirus (KSHV), vaccinia virus (VACV), adenovirus (AD) and influenza A virus (IAV) (12, 13, 14). Normal healthy cells typically utilize glucose for their bioenergetic needs whereas cancer cells often reprogram their metabolism by increasing the utilization of glutamine as the primary nutrient to support their TCA cycle and satisfy the metabolic requirements necessary for their high rates of proliferation and ability to survive various types of cellular stress (15, 16, 17, 18, 19, 20, 21). Members of the glutaminase family of enzymes play a critical role in satisfying these metabolic requirements by catalyzing the first step in glutamine metabolism, the hydrolysis of glutamine to glutamate, with glutamate then being converted to α-ketoglutarate by glutamate dehydrogenase to enter the TCA cycle (21, 22).

Two genes, *Gls* and *Gls2*, encode the glutaminase enzymes in mammals (16). *Gls* encodes KGA (kidney-type glutaminase) and the C-terminal truncated splice variant GAC (glutaminase C), herein collectively referred to as GLS, which are ubiquitously expressed in mammalian tissues, while *Gls2* encodes LGA (liver-type glutaminase, hereon designated GLS2) and is primarily but not exclusively expressed in liver, pancreas, and brain (23, 24, 25, 26). GLS is highly expressed in various types of cancers including basal-subtype triple-negative breast cancer, glioblastoma, and pancreatic cancers (27, 28), due to the actions of the transcription factors c-Myc and c-Jun (29, 30). GLS2 was recently implicated in luminal subtype breast cancer (28, 31). We and others have reported that allosteric inhibitors of GLS, including 968, BPTES, CB839, and UPGL00004 (designated as UP4 from hereon) block cancer cell proliferation (25), (32), with CB839 being examined in a number of clinical trials as an anti-cancer drug (26, 33, 34).

GLS inhibitors including BPTES and CB839 have also been shown to block the replication of some viruses (13, 14), while the glutamine antagonist L-DON, which inhibits glutaminases, transglutaminase, and other glutamine-utilizing enzymes, was reported to inhibit SARS-CoV-2 infection in hamster astrocytes (35). However, thus far, it has not been demonstrated that the newer GLS allosteric inhibitors UP4 and SU1 are capable of blocking coronavirus replication, and much remains to be determined regarding the role of glutamine metabolism in coronavirus infection (36). Here we show that three members of the coronavirus family, SARS-CoV-2, HCoV-OC43, and HCoV-229E reprogram the metabolic machinery of host cells, and in doing so, cause their replication to become glutamine-addicted. In these studies, we have used different cell lines for each coronavirus based on the expression of viral binding receptors; the mouse kidney epithelial cell line VeroE6 for SARS-CoV-2 infections, the human bronchial epithelial cell line HBEC and human colon cancer HCT8 epithelial cells for HCoV-OC43, and the lung epithelial cell line MRC5 for HCoV-229E (Table 1). Finally, two newer compounds that we developed, a pan-glutaminase inhibitor SU1 and a GLS-selective compound UP4, were tested against SARS-CoV-2-induced morbidity and mortality in the K18-hACE2 transgenic mice model, where they were shown to significantly reduce infection while extending the survival of these animals.

**Table 1:** Human coronaviruses and host cells

Species	Genus	Used	Host cell
HCOV-229E	Alpha	Yes	MRC5
HCOV-NL63	Alpha	No	
HCOV-OC43	Beta	Yes	HCT8, HBEC
SARS-CoV-2	Beta	Yes	VeroE6
SARS-CoV	Beta	No	
HCOV-HKU1	Beta	No	
MERS-CoV	Beta	No	

There are seven human coronaviruses. The three viruses used for this study are listed with their host cells.

## Results

### Coronavirus infection induces metabolic reprogramming in host cells

**Figure 1:**
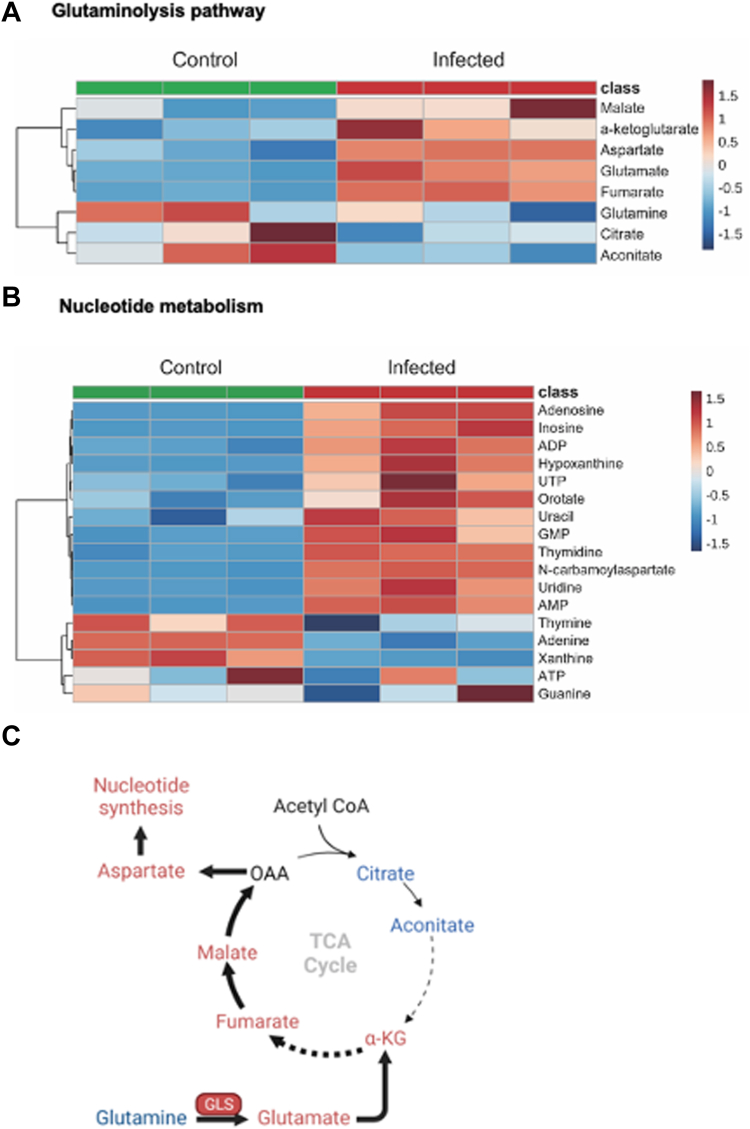
**Coronavirus infection induces metabolic reprogramming in host cells.***A*, HBECs were infected with HCoV-OC43 (MOI 0.01) for 24 h before metabolites were extracted. Related carbon metabolic pathways, including the TCA cycle, glycolysis, pentose phosphate pathway, and nucleotide synthetic pathways, were analyzed *via* targeted metabolomics. The heatmap showed the glutamine metabolic pathway components change before and after virus infection (n = 3 for each condition). *B*, heatmap of nucleotide metabolism components change before and after virus infection (n = 3 for each condition). *C*, the diagram shows the upregulated (in *red*) and downregulated (in *blue*) metabolites in HCoV-OC43-infected HBECs.

**Supporting Figure 1:**
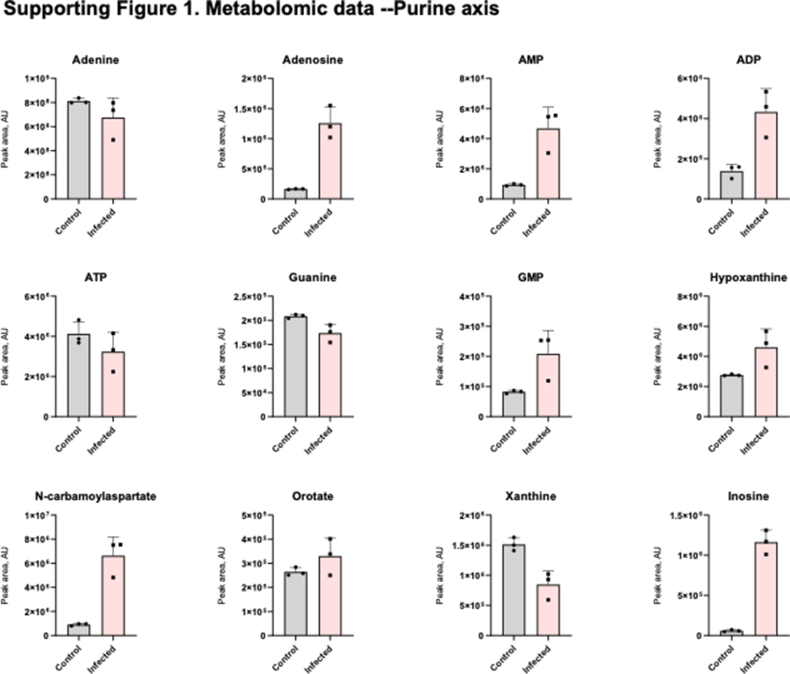


**Supporting Figure 2:**
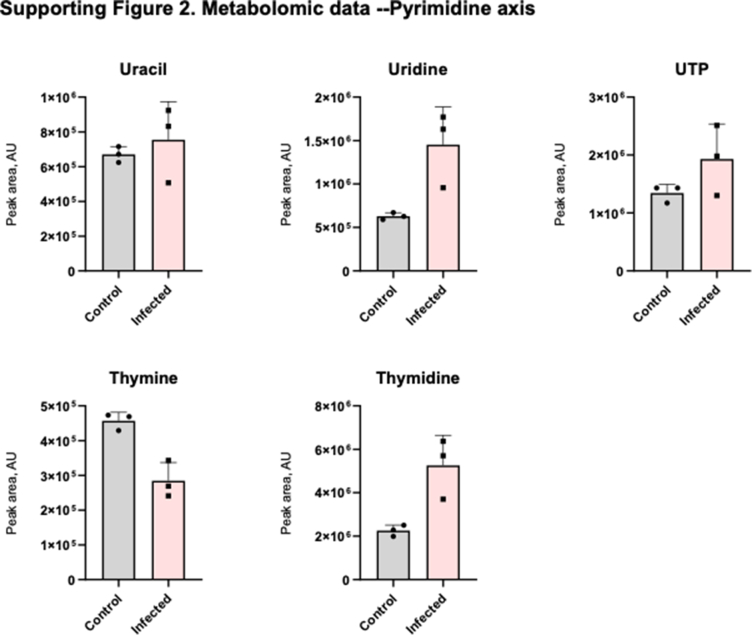


Viruses induce metabolic reprogramming in infected cells, and their replication depends on biosynthetic precursors and ATP provided by the host (37). Since glutamine supplies multiple biosynthetic pathways with carbon and/or nitrogen (13, 38), we were interested to see whether coronavirus infection upregulates glutamine metabolism in primary human bronchial epithelial cells HBEC3-KT (HBEC from hereon). We, therefore, applied LC-MS-based targeted metabolomics to metabolite extracts from HBECs that were either uninfected or infected for 24 h with the human beta-coronavirus HCoV-OC43. Upon infection, there was an apparent activation of glutamine metabolism, as read out by increases in the products of glutaminolysis, namely, glutamate and α-ketoglutarate, and downstream metabolites such as fumarate and aspartate, as well as several nucleotides (Fig. 1, *A*–*C*; also, Figs. S1 and S2), consistent with the known dependence of viral replication on activated nucleotide biosynthesis in host cells (39).

### Glutaminase expression is upregulated during coronavirus replication

**Figure 2:**
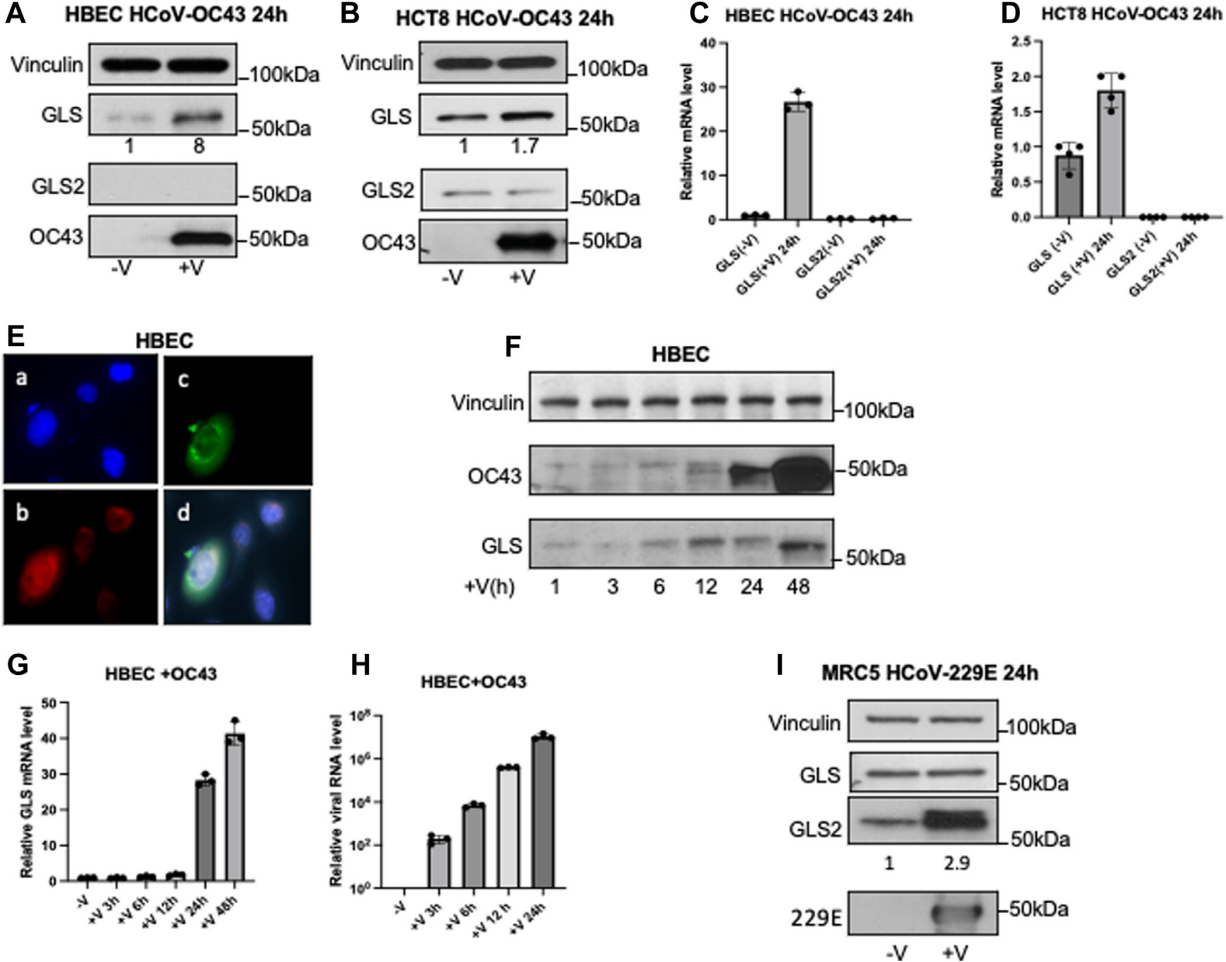
**GLS expression is increased during coronavirus infection.***A*, Whole-cell lysates of uninfected HBECs or HBECs infected with HCoV-OC43 (MOI 0.01, 24 h) were analyzed for viral N-protein OC43, GLS, and GLS2 expression levels by Western blot. *B*, similar experiment as (*A*) in HCT8 cells. *C*, total RNA was isolated from uninfected HBECs or HBECs infected with HCoV-OC43 (MOI 0.01, 24 h). qPCR assays showed GLS and GLS2 mRNA levels before and after HCoV-OC43 infections (n = 3). *D*, qPCR assays performed in HCT8 cells as in (*C*) (n = 4). *E*, co-immunofluorescent staining with HCoV-OC43 and GLS antibodies in HBECs. Cells were grown in 4-well slide chambers for 24 h and infected with the HCoV-OC43 virus for 24 h. One drop per well of NucBlue Live Cell stain solution was added to each well before the cells were fixed with formaldehyde (3.7%) (a) nuclear staining with DAPI (*blue*), (b) staining with GLS rabbit polyclonal antibody and goat anti-rabbit Alexa Fluor 568 secondary antibody (*red*), (c) staining with HCoV-OC43 mouse monoclonal antibody and goat anti-mouse Alexa Fluor 488 secondary antibody (*green*). (d) Merging of the images described in a,b,c. *F*, Western blot analysis shows the time course of GLS and HCoV-OC43 expression levels during virus infection in HBECs. *G*, qPCR assays for GLS levels were performed on total RNA samples isolated at different time points of virus-infected HBECs (n = 3). *H*, qPCR assays for HCoV-OC43 levels were performed on the total RNA samples isolated at different time points of virus-infected media in HBECs (n = 3). *I*, whole cell lysates of MRC5 cells uninfected or infected with HCoV-229E (MOI 0.01, 24h) were analyzed by Western blot for GLS and GLS2 expression levels.

Based upon the changes in glutamine metabolism caused by coronavirus infection, we examined whether glutaminase expression was upregulated when host cells were infected with HCoV-OC43. HBECs and HCT8 cells, when 80% confluent, were infected with HCoV-OC43 for 24 h, and then the cells and their medium were collected. Western blot analyses were performed using an anti-coronavirus antibody, OC43 strain to detect the HCoV-OC43 level as a read-out for viral replication, and anti-GLS and anti-GLS2 antibodies were used to identify the two forms of glutaminase expressed in the human epithelial cells (Fig. 2*A*). Although GLS protein expression was relatively low in HBECs, it increased significantly (eightfold) upon HCoV-OC43 infection. On the other hand, GLS2 protein expression was not detected before or after virus infection, indicating that only GLS is involved in coronavirus HCoV-OC43 replication in HBECs. In HCT8 cells, GLS protein expression was modestly increased (1.7-fold) with virus infection, whereas GLS2 protein levels were not changed (Fig. 2*B*). However, the basal levels of both GLS and GLS2 were higher in HCT8 cells compared to HBECs, most likely because the former represents a human colon cancer cell line. Quantitative PCR (qPCR) assays showed that the RNA transcript levels of GLS but not GLS2 were upregulated in both virus-infected HBECs and HCT8 cells (Fig. 2, *C* and *D*).

We then carried out immunofluorescence experiments in HBECs to visualize GLS expression as a function of virus infection at a cellular level. Cells were cultured in a 4-well chamber slide with 60% to 70% confluency, infected the next day with HCoV-OC43 as described earlier for 1 h, and then incubated in fresh culture medium for 22 h with nuclear staining for 1 h with NucBlue. After fixation, immunofluorescence staining was carried out using antibodies specific for either GLS or the HCoV-OC43 N protein, with the cell nuclei being visualized. Four HBECs shown in (Fig. 2*E*-a blue) exhibited different levels of GLS (Fig. 2*E*-b, red) and HCoV-OC43 expression (Fig. 2*E*-c, green). The merged images show that the virus-infected cells consistently expressed higher levels of GLS (Fig. 2*E*-d).

Next, we performed a time course for HCoV-OC43 infection of HBECs. The cells (70–80% confluent) were infected with HCoV-OC43 for 1 h, with the cells from one plate then being collected. Fresh medium was added to the remaining plates, and additional samples of cells and their culture medium were collected after 3, 6, 12, 24, and 48 h of incubation at 37 °C. The expression levels of the HCoV-OC43 nucleoprotein and GLS were detected by Western blot analyses from whole cell lysates (Fig. 2*F*), while qPCR was used to measure the total RNA transcript levels of GLS (Fig. 2*G*) and of viral RNA in the media (Fig. 2*H*). Both OC43 and GLS expression levels in HBECs showed significant increases between 12 and 48 h of virus infection.

We also examined glutaminase expression in MRC5 cells infected by HCoV-229E (analpha coronavirus). MRC5 cells at 80% confluency were infected with HCoV-229E (MOI 0.01, 1 h, at 33 °C), and then incubated for 23 h with growth medium at 37 °C. Western blot analyses of whole cell lysates showed that GLS2 expression was increased but not GLS (Fig. 2*I*).

### GLS is essential for coronavirus replication in HBECs

**Figure 3:**
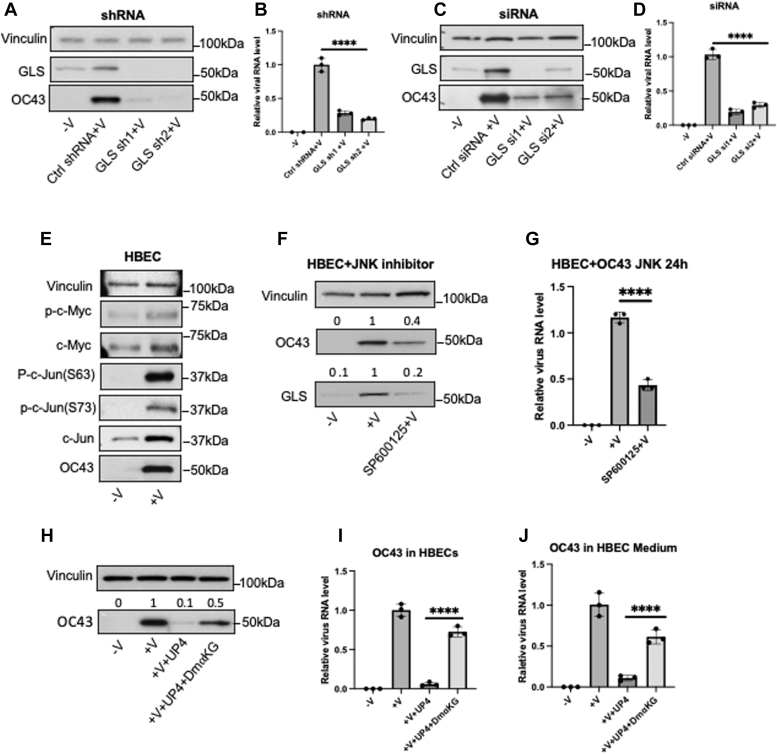
**GLS is essential for coronavirus replication in HBECs.***A*, Western blot analysis showing HCoV-OC43 replication levels in HBECs expressing a control shRNA or two independent GLS-targeted shRNAs. *B*, qPCR assays showing the relative HCoV-OC43 RNA levels in the media of control and two GLS-targeted shRNAs. *C*, Western blot analysis showing HCoV-OC43 replication levels in HBECs expressing control or two GLS-targeted siRNAs. *D*, qPCR analysis showing the relative HCoV-OC43 virus RNA levels from the media of non-virus infection, virus-infected cells with control siRNA, and two GLS-targeted siRNA knockdowns. *E*, HBECs were infected with HCoV-OC43 for 24 h (described previously) with the non-infected cells as a control. The whole cell lysates were collected. Western blot analysis shows that p-c-Jun(S73), p-c-Jun(S63), c-Jun, p-c-Myc and c-Myc levels are upregulated upon HCoV-OC43 infection of HBECs. *F*, Western blot data showing that blocking c-Jun activation by inhibiting the c-Jun-N-Terminal kinase (JNK) with inhibitor SP600125 (10μM) for 24 h reduces GLS expression in virus-infected host cells. *G*, qPCR assays showing the relative HCoV-OC43 mRNA levels in the media from cells treated with or without JNK inhibitor. (H) HBECs were infected with HCoV-OC43 (MOI 0.01) for 1 h compared with the noninfected cells, then the cells were treated with UP4 (1μM) and with or without Dimethyl-2-Ketoglutarate (5mM). The cells and the media were collected after 23 h of incubation. Western blot analysis of the cell lysates shows that Dimethyl-2-Ketoglutarate can rescue the OC43 replication inhibited by UP4. *I*, qPCR assays from the total RNA isolated from the cells for the same treatment conditions as (*H*). *J*, qPCR assays from the RNA isolated from the media for the same treatment conditions as (*H*). One-way ANOVA with Bonferroni correction was used to determine significance in *B*, *D* and *G*, ∗∗∗∗ indicates modified *p* < 0.0001.

To determine whether GLS expression is necessary for coronavirus infection, we knocked down GLS in HBECs for 24 h, using two different shRNAs. The cells were then infected with HCoV-OC43 for 24 h, and cell lysates were analyzed by Western blotting to determine GLS expression, while their medium was collected and analyzed by qPCR to detect total viral RNA. We found that HCoV-OC43 replication was reduced significantly when GLS was depleted from the cells, as evidenced by the decreased expression of the viral protein OC43 (Fig. 3*A*) and the reduced levels of viral RNA transcripts in the medium (72% and 80%, respectively; Fig. 3*B*). Similar reductions in HCoV-OC43 protein expression (Fig. 3*C*), and the levels of viral RNA transcripts in the medium (Fig. 3*D*) were observed when using two independent siRNAs targeting GLS.

We then wanted to know whether α-ketoglutarate, the product of glutamate dehydrogenase downstream from glutaminase in the glutamine metabolic pathway, could overcome the inhibition of viral replication. HBECs were infected with the HCoV-OC43 viruses for 1 hour at 33 °C. The infected cells were treated with DMSO, UP4 (1μM), or a combination of UP4 (1μM) and Dimethyl-2-Ketoglutarate (5mM) for 23 h. The cells and media were collected, followed by Western blot and qPCR analyses of the cell lysates (Fig. 3, *H* and *I*) and the media (Fig. 3*J*) for the different treatment conditions. We found that Dimethyl-2-Ketoglutarate can rescue the virus replication inhibited by the UP4 compound.

### GLS expression is upregulated in HBECs by c-Jun during virus infection

GLS expression in cancer cells has been reported to be upregulated either by c-Myc or c-Jun (29, 30), and the JNK/c-Jun pathway is activated in some infected cells (40, 41, 42, 43). To further examine how GLS expression is increased upon virus infection, HBECs were infected with HCoV-OC43 for 1 hour followed by a 23-h incubation, at which point the cells were collected and Western blot analyses performed. Phospho-c-Myc, phospho-c-Jun(S63), and phospho-c-Jun(S73) were all observed to be increased after infection, as were the total protein expression levels for c-Myc and c-Jun together with the viral protein OC43 (Fig. 3*E*). However, while inhibiting c-Myc did not cause a reduction in the expression levels of GLS in virus-infected host cells (HCT8) (not shown), blocking c-Jun activation using the small molecule inhibitor SP600125 reduced both GLS protein expression and the amount of viral RNA transcripts detected in the medium (Fig. 3, *F* and *G*, respectively).

### Glutaminase inhibitors do not prevent coronaviruses from entering cells but block their replication

**Figure 4:**
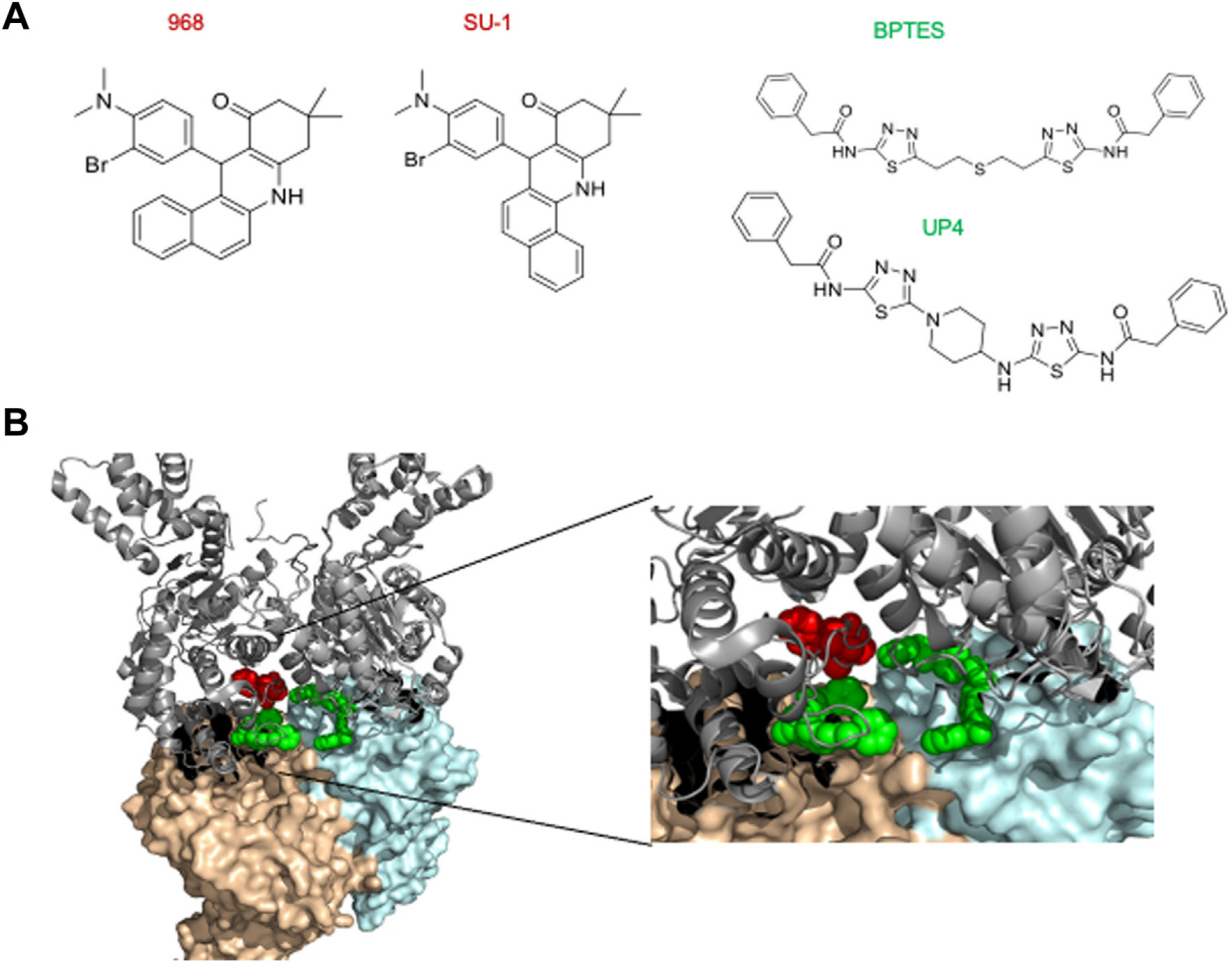
**Allosteric inhibitors targeting glutaminase.***A*, the structures of two classes of glutaminase inhibitors: 968 and the more potent analog SU1, and BPTES and its more potent analog UP4. *B*, the homo-tetrameric structure of human GLS shows the proposed binding site for 968 and SU1 (*red* color) based on a recent cryo-EM structure for the 968-GLS complex, and the binding location for the BPTES and UP4 compounds (*green*) as determined by X-ray crystallography.

We and others have developed and characterized two classes of allosteric glutaminase inhibitors, based on the lead compounds 968 and BPTES (27, 44, 45, 46, 47) (Fig. 4*A*). The 968 class of molecules are pan-glutaminase inhibitors as they inhibit both GLS and GLS2, whereas the BPTES family of compounds is selective for GLS. X-ray crystal structures have shown that BPTES, and its more potent analogs CB839 and our newly developed inhibitor UP4, bind in the interface where two dimers of GLS come together to form a tetramer (48), while 968 and the more potent SU1 compound appear to bind in close proximity but not directly overlapping the BPTES/CB839/UP4 binding sites (Fig. 4*B*) (45). Both classes of inhibitors trap the glutaminase enzymes in an inactive tetrameric state, although the 968 group of compounds also induces the formation of some inactive dimer species (45).

**Supporting Figure 3:**
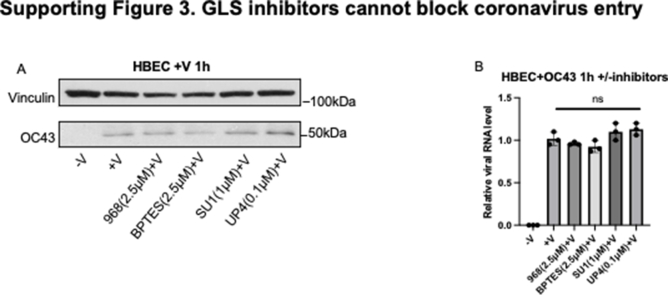


We first tested whether GLS inhibitors block coronaviruses from entering cells. The entry of coronaviruses into their host cells depends upon binding to membrane-associated receptors. HBECs lack the ACE2 receptor and thus we did not observe these cells to be infected by SARS-CoV-2. However, HCoV-OC43 can infect HBECs as well as HCT8 cells (Fig. 2, *A*–*D*). HBECs cells were pretreated with either 968, BPTES, SU1 or UP4 or untreated for 3 h. The cells were infected with HCoV-OC43 (MOI 0.01, 2%FBS in RPMI) for 1 h at 33 °C, at which point virus replication was minimal, and then collected. Western blot analyses were performed to detect the OC43 viral protein level in the cell (Fig. S3*A*). We found that OC43 was present under all conditions of inhibitor treatments. The presence of viral RNA in the cells as determined by qPCR was also unaffected by the inhibitors (Fig. S3*B*).

**Figure 5:**
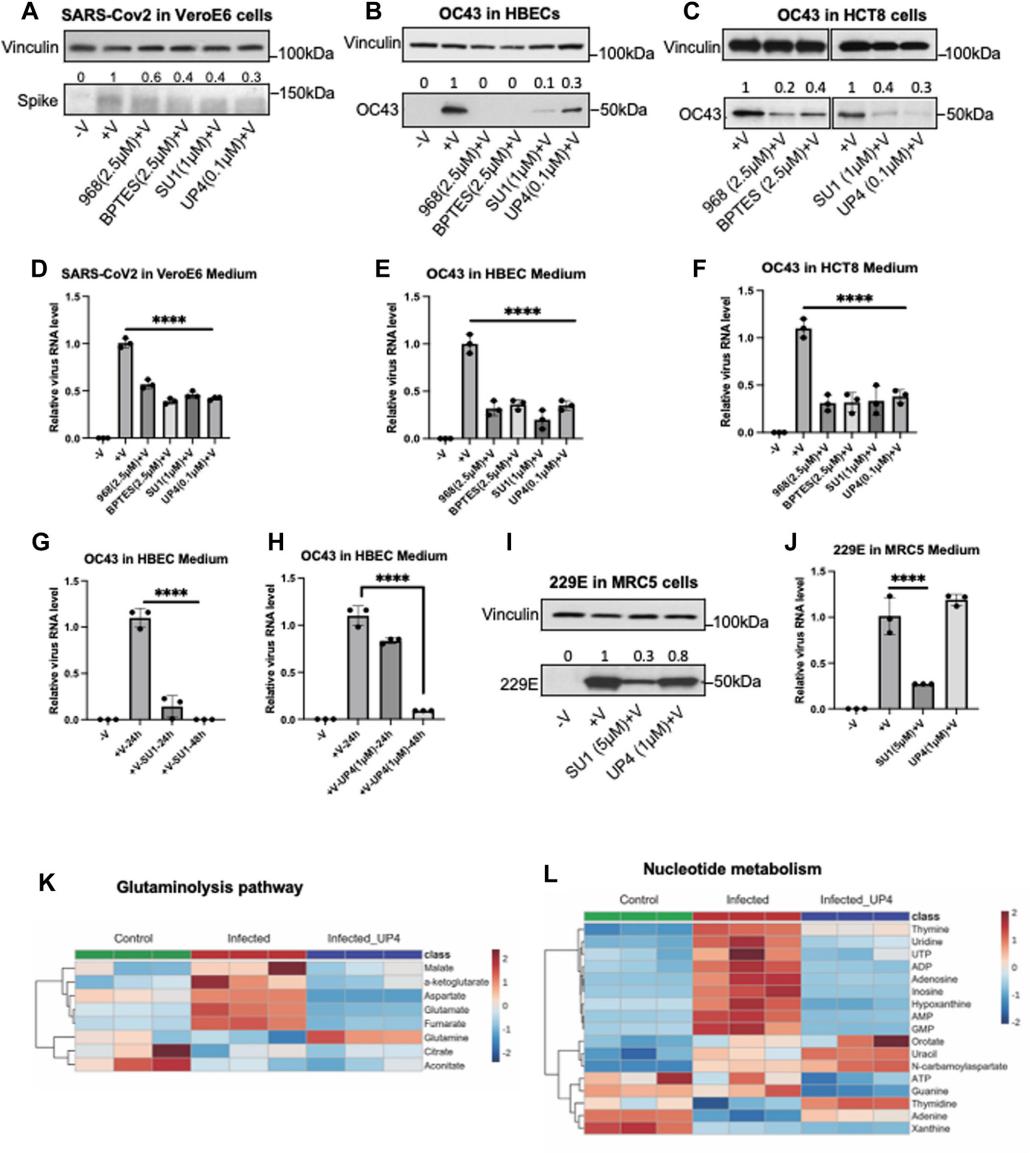
**Glutaminase inhibitors block coronavirus SARS-CoV-2, HCoV-OC43 and HCoV-229E replication.***A*, VeroE6 cells when 70 to 80% confluence were pretreated with 968 (2.5μM), BPTES (2.5μM), SU1 (1μM), UP4 (0.1μM), and vehicle control (DMSO) for 3 h, followed by SARS-CoV-2 infection (MOI 0.01) for 1 h at 37 °C. The growth medium of each condition was changed with or without inhibitors and the cells were incubated at 37 °C for 23 h. The media and cells were collected. Western blot analysis of the inactivated whole cell lysates showing the SARS-CoV-2 replication levels in cells treated with the different inhibitors compared with DMSO treated virus-infected cells. *B*, HBECs were pretreated with DMSO, 968 (2.5μM), BPTES (2.5μM), SU1 (1μM), and UP4 (0.1μM) for 3 h, followed by HCoV-OC43 infection for 1 h at 33 °C (or no infection), and then incubated in growth medium with or without inhibitors for 23 h at 37 °C. Western blot analysis shows the HCoV-OC43 replication levels in cells treated with different inhibitors compared with non-treatment. *C*, HCT8 cells were pretreated with HCoV-OC43 and inhibitors as in (*B*) and analyzed for virus replication by Western blot. *D*, qPCR assays showing the relative virus RNA levels in the SARS-CoV-2 infected VeroE6 cell media with or without inhibitors. *E*, qPCR analysis showing the relative virus RNA levels in the media of HCoV-OC43 infected HBECs with or without inhibitors (n = 3 for each condition). *F*, relative HCoV-OC43 virus RNA levels from the media of HCT8 cells treated with or without inhibitors and infected with or without HCoV-OC43 (n = 3 per condition). (*G*) HBECs were infected with HCoV-OC43 for 1 h or were not infected, followed by incubation with growth medium for 2 h, and then the infected cells were treated with or without SU1(5μM) for 21 h or 45 h. The media were collected and followed by qPCR assay. *H*, the same experiment as in (*G*) was performed with UP4 (1μM) treatment for 21 h or 45 h. The qPCR assays were performed under different media conditions. *I*, MRC5 cells were pretreated with DMSO, SU1 (5μM) and UP4 (1μM) for 3 h, followed by HCoV-229E infection for 1 h at 33 °C. The growth medium was changed to that for the pretreated condition for 23 h and then the cells and medium were collected. Western blot assays of the cell lysates showed the 229E virus levels for the different conditions. (*J*) qPCR assays showing the relative viral-RNA levels of MRC5 infected with 229E media with or without GLS inhibitors (n = 3 per condition). *K*, HBECs pre- and post-treated with UP4 (1μM) were infected with HCoV-OC43 (MOI 0.01) for 1 h and were continuously incubated for 23 h before metabolites were extracted (n = 3 for each condition). Related carbon metabolic pathways were analyzed *via* targeted metabolomics. The heatmap showed glutamine metabolism pathway component changes with or without UP4 treatment during virus infection compared with wildtype cells. *L*, the heatmap of nucleotide metabolism components before and after virus infection or for virus infected cells treated with UP4 (n = 3 for each condition). One-way ANOVA with Bonferroni correction was used to determine significance in *D*, *E*, *F* and *H*, ∗∗∗∗ indicates modified *p* < 0.0001.

**Supporting Figure 4:**
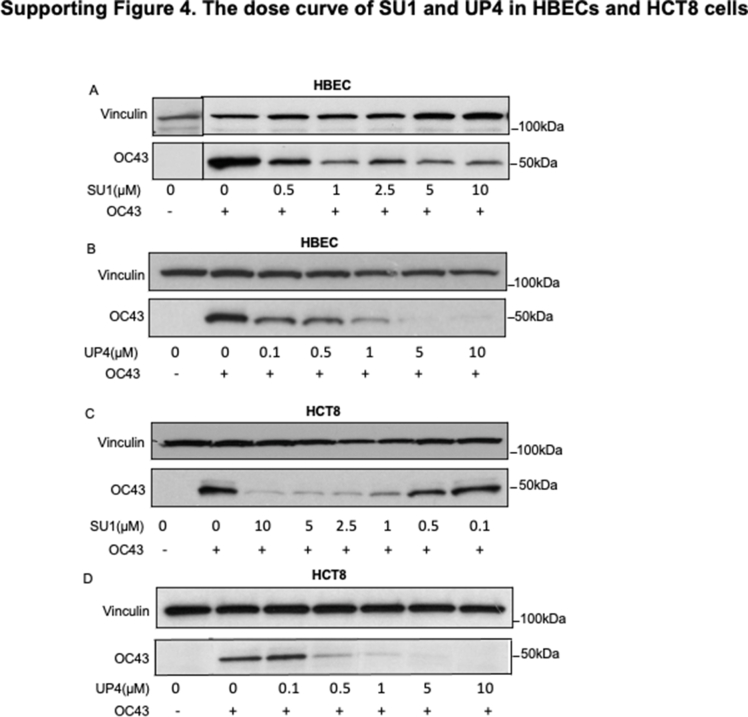


**Supporting Figure 5:**
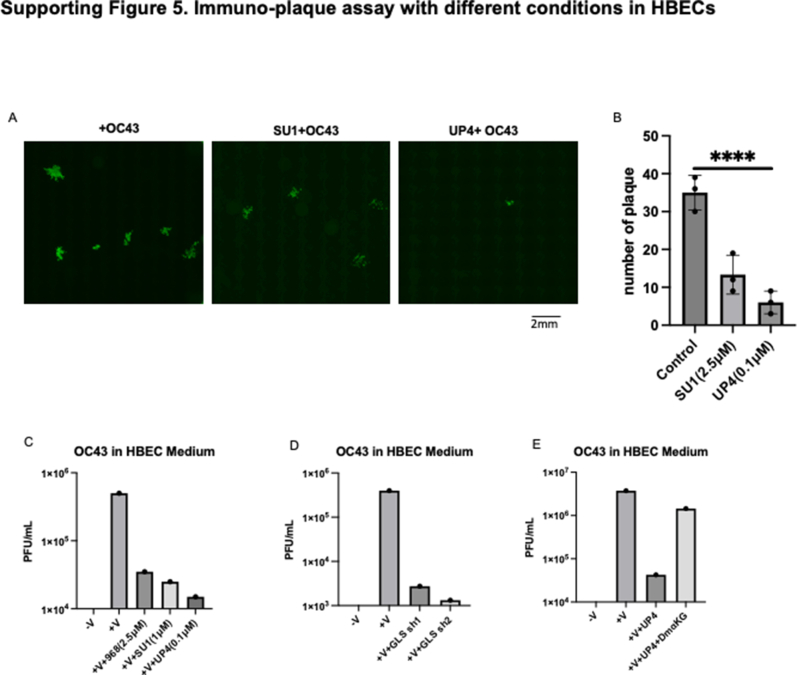


We then examined the ability of small-molecule glutaminase inhibitors to block coronavirus replication. In these studies, we examined HCoV-229E which belongs to the alpha-genus of the coronavirus family, and SARS-CoV-2 and HCoV-OC43 from the beta-genus (Table 1). In one set of experiments, cells were grown to 70 to 80% confluence and then pretreated for 3 h with a glutaminase inhibitor (either the pan-GLS/GLS2 inhibitors 968 or SU1 or the GLS selective inhibitors BPTES or UP4), or with vehicle control, followed by infection of VeroE6 cells with SARS-CoV-2 (MOI 0.01, 2%FBS in DMEM) for 1 h at 37 °C, and HBECs or HCT8 cells with HCoV-OC43, (MOI 0.01, 2% FBS in RPMI medium) for 1 h at 33 °C. Following a 23-h incubation at 37 °C, the cells and their media were collected, and Western blot analyses were performed on the cell lysates to detect the total levels of the SARS-CoV-2 (Fig. 5*A*) and HCoV-OC43 proteins (Fig. 5, *B* and *C*). We observed that virus replication of SARS-CoV-2 and HCoV-OC43 was significantly suppressed by the glutaminase inhibitors in a dose-dependent manner, as read out by reductions in the expression of the SARS-CoV-2 spike protein and the HCoV-OC43 N protein (Figs. 5, *A*–*C* and S4, *A*–*D*). We confirmed these results by plaque assays (Fig. S5, *A* and *B*) and by qPCR measurements of the virus RNA levels for SARS-CoV-2 in the VeroE6 medium (Fig. 5*D*) and the HCoV-OC43 RNA levels in both HBEC (Fig. 5*E*) and HCT8 media (Fig. 5*F*).

We also examined the effectiveness of a post-treatment of HCoV-OC43-infected HBECs with the glutaminase inhibitors SU1 and UP4. HBECs were infected with HCoV-OC43 (MOI 0.01) for 1 h at 33 °C and then incubated with HBEC growth medium at 37 °C for 2 h. At that point, the infected cells were either treated with vehicle control, SU1 (5μM), or UP4 (1μM), for 24 and 48 h. The media were collected, and total virus RNA was isolated, followed by qPCR analysis. The virus level was significantly reduced when cells were treated with SU1 for 24 h post-infection (Fig. 5*G*), whereas UP4 was less effective. However, when treated for 48 h following viral infection, it caused a significant reduction in the amount of virus shed into the media (Fig. 5*H*).

Interestingly, we observed a highly specific effect by SU1 when experiments were performed with MRC5 cells infected with HCoV-229E. In these experiments, MRC5 cells were pretreated with DMSO, SU1 (5μM), and UP4 (1 μM) for 3 h, and then infected with HCoV-229E (MOI 0.01, 1 h at 33 °C), followed by an incubation at 37 °C with fresh culture medium containing either DMSO, or SU1 or UP4 for 23 h. The cells and media were collected, followed by Western blot and qPCR analyses. Here we found that the pan-GLS/GLS2 inhibitor SU1 was highly effective at blocking HCoV-229E replication, while the GLS-specific compound UP4 was relatively ineffective (Fig. 5, *I* and *J*), consistent with our findings that infection of MRC5 cells predominantly upregulates the GLS2 isoform (Fig. 2*I*).

### Glutaminase inhibition reverses coronavirus-induced metabolic changes

**Supporting Figure 6:**
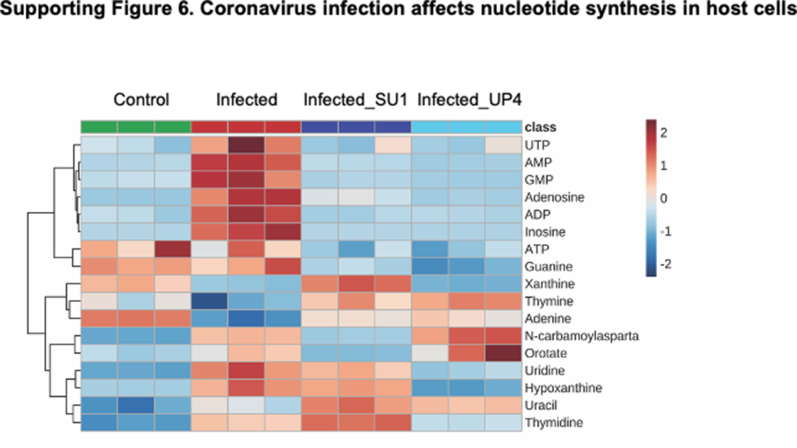


Next, we examined how inhibiting glutaminase activity affects coronavirus-induced metabolic reprogramming in host cells. HBECs were infected with HCoV-OC43, whose viral replication was sensitive to both classes of glutaminase inhibitors. The cells were infected for 24 h in the absence or presence of 1 μM UP4, and cell extracts were then prepared for targeted metabolomics analysis. Heatmaps of glutamine and nucleotide metabolism of the three groups of HBECs, non-infected, infected with HCoV-OC43, or infected with HCoV-OC43 in the presence of UP4 (Fig. 5, *K* and *L*). As expected, treatment with UP4 abolished the increases in glutamate, downstream TCA cycle intermediates, and aspartate during HCoV-OC43 infection (Fig. 5*K*), as well as those of nucleosides and nucleotides (Fig. 5*J*) and similar results were obtained with SU1 (Fig. S6).

### Glutaminase inhibitors increase the survival of SARS-CoV-2-infected mice

**Figure 6:**
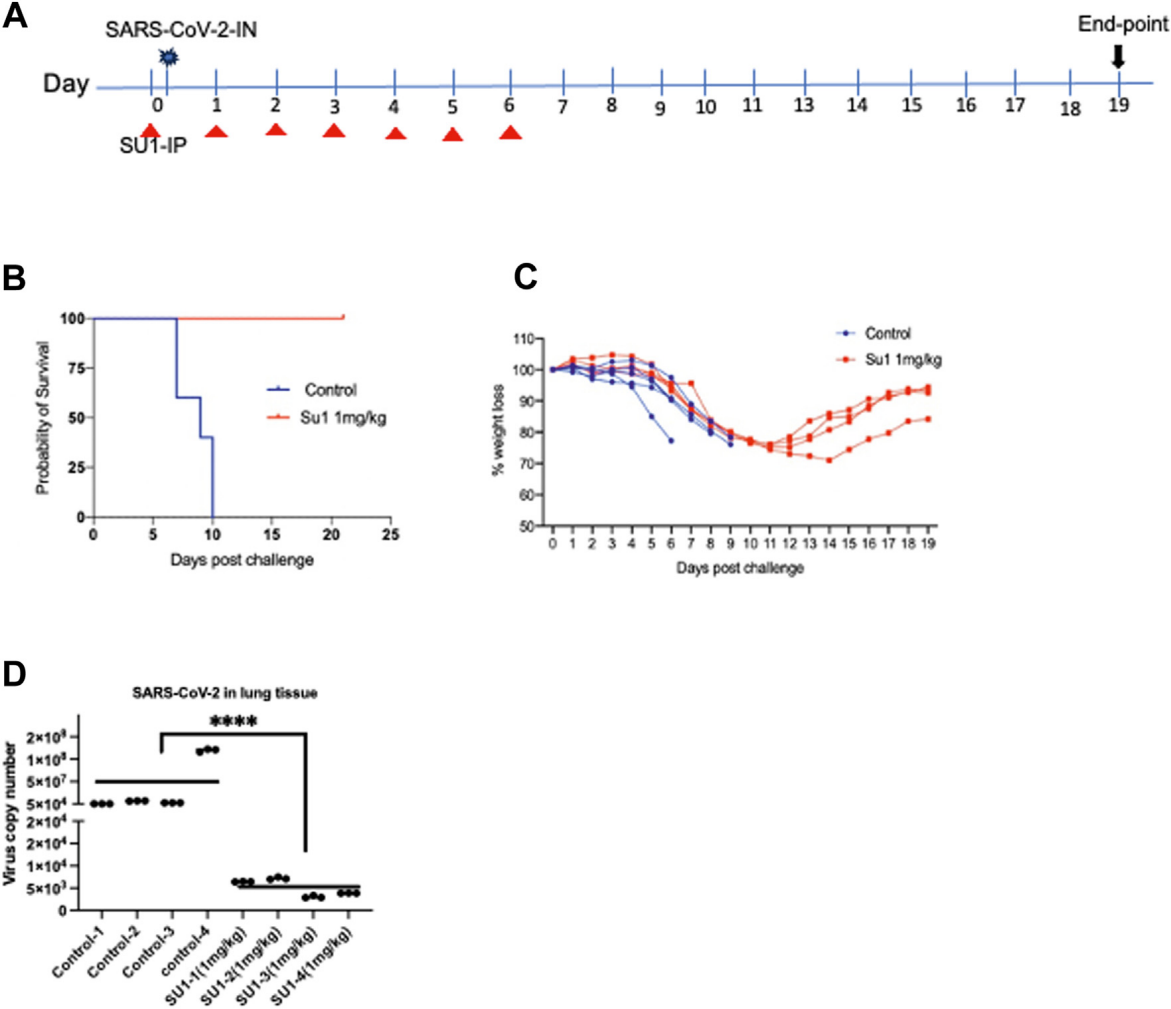
**SU1 provides a survival benefit to SARS-CoV-2-infected mice.***A*, K18-hACE2 mice (n = 4 per treatment group) were pre-treated with inhibitors by intraperitoneal (IP) injection 3 h before virus infection, followed by intranasal (IN) infection with 2 × 10^3^ PFU of SARS-CoV-2 (WA1 2020) at day 0. The mice were treated with SU1 (1mg/kg) daily through IP injection from days 1 to 6. Surviving mice were collected on day 19. *B*, probability of survival. *C*, weight change of control and SU1 treated mice. *D*, a qPCR assay was performed on the lung tissues of the mice (n = 4). One-way ANOVA with Bonferroni correction was used to determine significance in D, ∗∗∗∗ indicates modified *p* < 0.0001.

**Supporting Figure 7:**
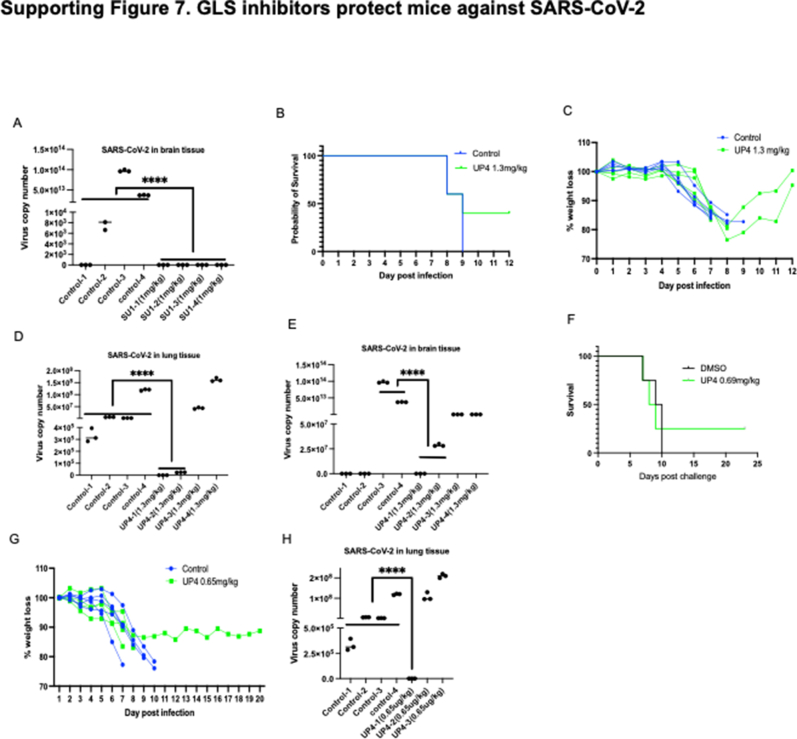


We then investigated whether SU1 and UP4 inhibitors would improve morbidity and survival in SARS-CoV-2-infected K18-hACE2 transgenic mice (49, 50), an established mouse model of severe COVID-19 (51). We pre-treated each group of mice with either an inhibitor vehicle control or SU1(1mg/kg) 3 hours before the animals were challenged with 2 × 10^3^ PFU of SARS-CoV-2 (WA1/2020 variant) through intranasal administration (IN). The drug treatment was followed daily and delivered through intraperitoneal injection (IP) with 100 μl of a mixture containing 5% DMSO with or without, 5% ethanol, and 5% Cremophor EL. Our results showed that SU1(1mg/kg) treated SARS-CoV-2- infected mice all survived (n = 4) until they were collected at day 19, whereas the untreated control mice died within 10 days (n = 5) (Fig. 6*B*). We also tracked the weight changes for each group of animals. Three of the SU1 treated mice exhibited weight loss from day 5 to day 11, while another showed weight loss until day 14; however, each of the SU1 treated animals fully regained their weight. The control mice began to lose weight on day 5 and died within 9 days (Fig. 6*C*). We collected lung and brain tissues from the animals, and the total RNA from each sample was isolated. qPCR data showed that all four SARS-CoV-2 infected mice treated with SU1 exhibited significantly lower virus levels in both their lungs and brains, compared to the untreated control mice (Figs. 6*D* and S7*A*).

We also examined different doses of UP4 when treating the SARS-CoV-2 infected mice as described above. Two of the four mice treated with UP4 (1.3 mg/kg) survived (Fig. S7*B*) and regained their weight *i.e.*, 95% in one case and 100% in the other (Fig. S7*C*). The qPCR data showed that the SARS-CoV-2 virus numbers in lungs and brains of the mice that survived were significantly lower than in the control mice (Fig. S7, *D* and *E*). One of the four mice recovered from SARS-CoV-2 infection when treated with a lower dose of UP4 (0.65 mg/kg) (Fig. S7*F*), while exhibiting a weight loss of ∼15% (Fig. S7*G*). The qPCR data from lung tissues showed that the SARS-CoV-2 virus level in this mouse was undetectable (Fig. S7*H*).

## Discussion

The glutaminase family of mitochondrial metabolic enzymes has been shown to play important roles in cancer progression due to the ability of its members to catalyze the first step in glutamine metabolism, the hydrolysis of glutamine to glutamate with the accompanying production of ammonia (18, 19, 23, 25, 30, 52). By increasing glutaminolysis, cancer cells satisfy their metabolic requirements and glutamine addiction, which are an outcome of the Warburg effect that uncouples the glycolytic pathway from the TCA cycle. The elevations in glutamine metabolism that occur in cancer cells provide the carbon sources necessary to generate building blocks for biosynthetic processes that underlie their malignant phenotypes. It has been reported that some virus-infected host cells appear to undergo a reprogramming of their metabolism similar to cancer cells (21, 35). Here we show that this is the case for coronavirus infection including SARS-CoV-2 by demonstrating that glutamine metabolism is elevated in virus infected host cells and essential for viral replication.

There are two major forms of glutaminase enzymes in mammals and humans, designated here as GLS and GLS2. Increases in GLS expression and its specific activity have been implicated in several human cancers, although GLS2 has also been shown to be important in luminal-subtype breast cancer (28). In our studies, we have examined four different host cell lines and three members of the coronavirus family and found that in most cases GLS is essential for viral replication. However, in one host cell, MRC5, it appears that GLS2 is the glutaminase enzyme required for viral replication. The upregulated expression of GLS in cancer cells is an outcome of either c-Myc blocking the inhibitory actions of a microRNA (29), or through signaling pathways that result in the activation of the transcription factor c-Jun (30). For host cells infected by coronaviruses, we have found that c-Jun activation upregulates GLS expression. Thus far, very little is known regarding how glutaminase activity is activated either in cancer or virus-infected cells. We and others have shown that both GLS and GLS2 activation requires that the enzymes undergo a transition from an inactive dimer to a tetramer and then ultimately to a higher-order filament (53, 54). The formation of activated glutaminase filaments requires both the binding of substrate and an anionic activator. Inorganic phosphate is commonly used to serve as an activator *in vitro*, although the concentrations required (50–100 mM) may not be achieved in most physiological settings and therefore, we are setting out to determine what type of metabolite or cofactor might serve this function in cells.

**Figure 7:**
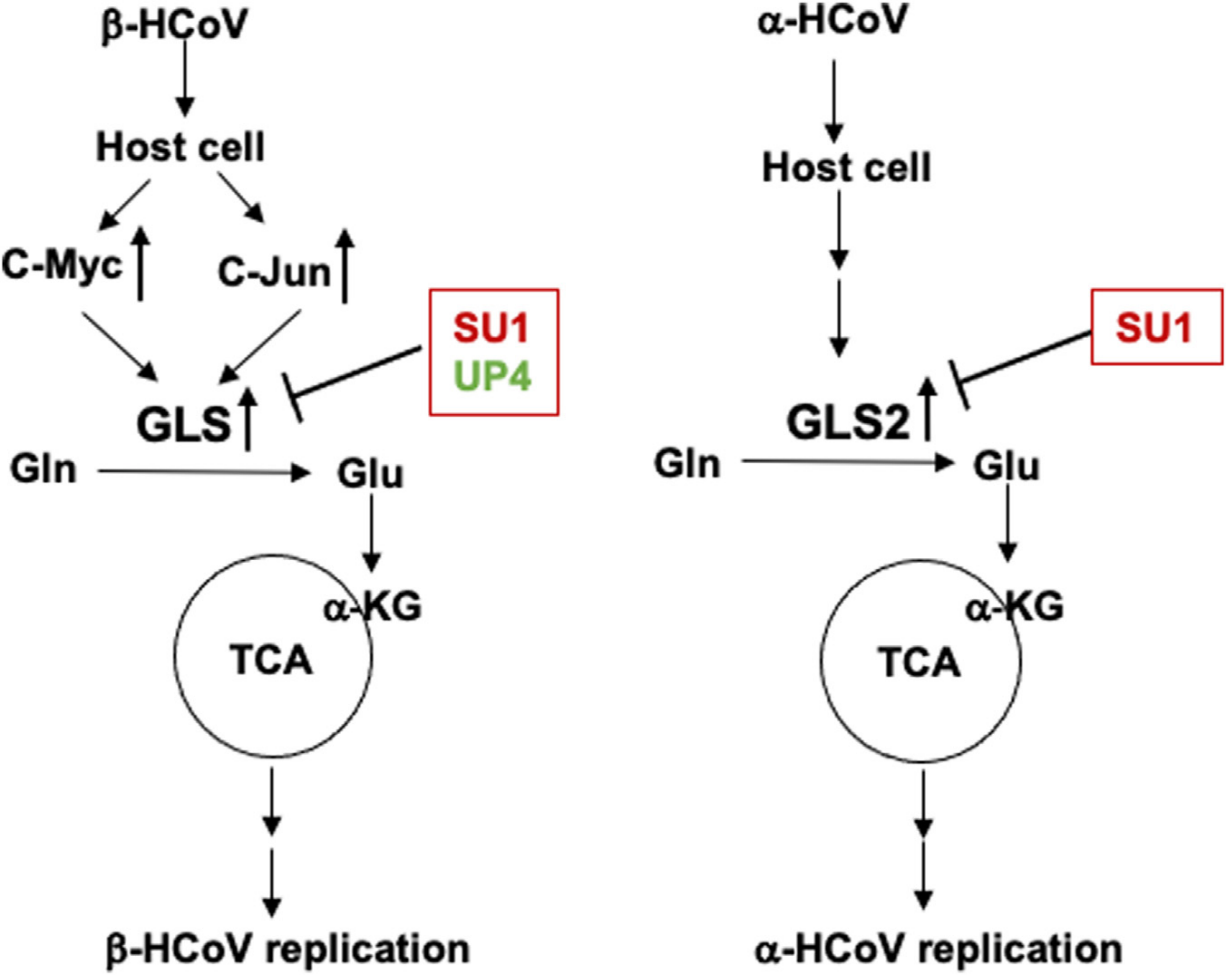
**Schematic of GLS and GLS2 mediated coronavirus replication.** Coronaviruses hijack the metabolic program of host cells by increasing the expression of glutaminase enzymes. Two possible mechanisms for upregulating GLS upon beta-coronavirus infection involve signaling through c-Myc and c-Jun. Alpha-coronavirus replication occurs by upregulating GLS2 expression (HCoV-229E in MRC5 cells).

There has been a significant amount of effort devoted to developing small molecule inhibitors that target the glutaminase enzymes, given their roles in tumorigenesis. Two of the more common types of GLS inhibitors are the 968 class of molecules and the BPTES family of inhibitory compounds (27, 44, 45, 46, 47, 55). Among the latter are CB839 and our more newly developed UP4 (48), which are significantly more potent than the lead compound BPTES, with CB839 being examined in clinical trials for various cancers (52, 56, 57). The BPTES series of compounds bind within the interface where two GLS dimers come together to form a tetramer and stabilize an inactive tetrameric species that is incapable of forming higher order filament-like structures (45). The 968 class of GLS inhibitors which includes the lead compound 968 and our more potent analog SU1 (46) function in a distinct manner from the BPTES class of compounds, by preferentially binding initially to GLS monomers and then stabilizing both inactive dimers and tetramers and preventing filament formation. Both classes of allosteric GLS inhibitors effectively blocked coronavirus replication in HBECs, HCT8, and VeroE6 cells, as read out by the synthesis of a viral coat protein and when assaying viral RNA transcript levels, matching the effects we observed when knocking down GLS expression in host cells. These inhibitors were effective either when pre-treating cells prior to virus infection or when added post-virus infection. In the case of MRC5 cells, only the 968 analog SU1 was effective at inhibiting viral replication. This is due to GLS2, which is relatively insensitive to UP4 and other related compounds (28), being highly upregulated upon virus infection of MRC5 cells, rather than GLS. These findings are summarized in the schematic presented in Figure 7.

Current approved anti-coronavirus drugs target proteins essential for virus replication, including RNA polymerase (58, 59, 60), spike proteins (59), proteases such as TMPRSS2 (6, 61), and nucleocapsid proteins (62). Our work has focused on blocking the metabolic activities required to generate building blocks for biosynthetic processes and the energy supply necessary for coronavirus replication. Because the glutaminase enzymes are often highly expressed and activated in cancer (17, 18, 20, 54) and viral-infected host cells (13, 14, 38, 63), as compared to normal healthy cells, small molecule inhibitors targeting these enzymes offer a potentially safe therapeutic strategy. Thus far, our experiments with SARS-CoV-2-infected K18-hACE2 mice suggest that glutaminase inhibitors can provide a significant survival advantage for infected animals. In these studies, the UP4 compound, which is a member of the BPTES subfamily of compounds that selectively inhibits GLS, was less effective than the pan-glutaminase inhibitor SU1. This may be due to UP4 being less well absorbed compared to SU1, or that it may accumulate in lipid membranes to a greater degree, which might also explain its reduced effectiveness in experiments when virus infected cells were treated with these inhibitors, post-infection. However, it is also possible that in some cases, GLS2, which is not effectively inhibited by UP4, will compensate for any UP4-mediated inhibition of GLS activity.

Our findings raise interesting questions and lines of investigation for future studies. They include developing a better formulation of glutaminase inhibitors and examining how broadly effective allosteric inhibitors of glutaminases are against other viruses, as well as determining how virus infection triggers the necessary signals to upregulate glutaminase expression. It also will be of interest to establish whether glutaminase filament-like structures form within virus-infected host cells, similar to cancer cells (45, 53, 64, 65). If so, do these higher order oligomeric structures serve as a scaffold for a metabolic complex necessary for satisfying the requirements for viral replication? While we recently showed that glutaminase inhibitors can block the ability of both GLS and GLS2 to form filaments (45), it will be interesting to see if additional strategies can be designed to specifically block their formation and thus yield additional classes of anti-viral therapeutics.

## Experimental procedures

### SARS-CoV-2 propagation and infection

Studies of SARS-CoV-2 were performed in a Biosafety Level 3 (BSL3) lab, and were approved by the Cornell University Institutional Biosafety and Animal Care and Use Committees. VeroE6 cells (Kidney epithelial cells isolated from African green monkey, ATCC, cat. CRL-1587) were grown in T75 flasks until 80 to 90% confluent. SARS-CoV-2 virus WA1 (BEI resources, cat. NR-52281) dilution was prepared in 2 ml of DMEM (Gibco), 2% FBS (Gibco) (MOI 0.1), per flask. After washing with PBS twice, virus dilution was added to the cell flasks for 1 h at 37 °C in 5%CO_2_ with continuous shaking, followed by adding 10 ml DMEM, 2%FBS medium, and incubating for 4 to 6 days or until the cytopathic effect (CPE) progressed through 80% of the cells. The virus medium was collected and centrifuged at 1200 rpm for 10 min. The supernatant containing the virus was aliquoted and stored at −80 °C in liquid Nitrogen. Plaque assays and qPCR were performed to determine the virus titer.

VeroE6 cells were grown in T75 flasks until 70% confluent, pre-treated with inhibitors for 24 h, and then infected with SARS-CoV-2 (MOI 0.01) for 1 h at 37 °C 5%CO_2_ with shaking. The plate media was changed to the growth medium with or without inhibitors and the incubation continued for 23 h at 37 °C, 5%CO_2._ The viral supernatant medium was inactivated by TRIzol (Thermo Fisher, cat. A33255), and used for RNA isolation and qPCR. The cells were collected, and cell lysates were prepared by adding RIPA buffer and then heat inactivated for Western blot assays.

### HCoV-OC43 propagation and infection

The HCoV-OC43 (ATCC, Betacoronavirus 1, cat. VR-1558) propagation followed the ATCC protocol. HCT8 (human colorectal carcinoma cell line initiated from an adult male, ATCC, cat. CCL-244) cells were grown in 15 cm plates until 80 to 90% confluent in RPMI (Gibco) containing 10% HS (horse serum, Gibco). Virus dilution was prepared in 7 ml of RPMI medium containing 2% HS (MOI 0.05). The monolayer cell plates were washed twice with PBS. Virus dilution was adsorbed by cells for 2 h at 33 °C, 5%CO_2_, in an incubator while shaking continuously. Ten ml of RPMI containing 2% HS medium were added and continued incubating for 4 to 6 days at 33 °C, 5%CO_2_, with shaking. The virus medium was collected when the CPE progressed through 80% of the monolayer and then was centrifuged at 1200 rpm for 10 min and aliquots of the supernatant containing the virus were stored at −80 °C. The virus titer was determined by plaque assay and qPCR analysis.

HCT8 cells or HBECs (Primary human bronchial epithelial cells, ATCC, cat. CRL-4051) were grown in 10 cm plates until 70 to 80% confluent at 37 °C, 5%CO_2_, and pretreated with inhibitors for 3 h. HCoVOC43 virus (MOI 0.01, RPMI 2% HS) was used to infect cells for 1 h at 33 °C, 5%CO_2_, with shaking. The different conditions of the growth medium of HCT8 cells or HBECs were changed as designed, and the cell plates were incubated at 37 °C, 5%CO_2_, for 23 h. The cells and media were collected for Western blot and qPCR analysis, respectively.

When treated with inhibitors post-infection, HBECs were grown in 10 cm plates until 70 to 80% confluent at 37 °C, 5% CO_2._ HBECs were infected with HCoVOC43 virus (MOI 0.01) for 1 h at 33 °C, 5% CO_2,_ with shaking. The HBECs growth medium was then changed and after 2 h switched to medium containing inhibitors, followed by incubations of 21 and 45 h. The media were collected for qPCR analysis.

### HCoV-229E virus propagation and infection

Following the ATCC protocol for HCoV-229E (Human coronavirus 229E, ATCC, cat. VR-740) virus propagation, MRC5 (normal human fetal lung fibroblast cells, ATCC, cat. CCL-171) cells were grown in EMEM, 10% FBS, 15 cm plates until 80 to 90% confluent. The virus dilution was prepared in 7 ml of EMEM with 2% FBS medium, MOI 0.05. The cells were adsorbed with a virus dilution at 35 °C, 5%CO_2_, with shaking for 2 h. Ten ml of EMEM with 2% FBS medium were added to the plates and incubated for 4 to 6 days until CPE progressed to 80%. The virus media were collected and centrifuged at 1200 rpm for 10 min. The supernatants containing viruses were aliquoted and stored at −80 °C. The virus titer was determined by qPCR and plaque assays.

MRC5 cells were grown for 70 to 80% confluent at 37 °C, 5%CO_2_, and were pretreated with the inhibitors for 3 h. They were then infected with the virus (MOI 0.01, EMEM 2% FBS) dilution for 1 h at 35 °C, 5%CO_2_, with shaking. The cell media were changed to the appropriate growth media conditions and the cells were incubated for 23 h at 37 °C, 5%CO_2_. The cells and media were collected for further analysis.

### Inhibitors and rescue compound

The glutaminase inhibitors SU1 and BPTES were synthesized by Dr. Scott Ulrich (Ithaca College). UP4 (UPGL00004) (Cat. SML2472) was originally developed by Dr. Lee McDermott in collaboration with the Cerione laboratory, and then obtained from Millipore Sigma. 968 was from ChemBridge Corporation; the c-Jun N-terminal kinase inhibitor SP600125 (Cat. S5567) and the c-Myc inhibitor 10,058-F4 (Cat. F3680) were from Millipore Sigma. Dimethyl-2-Ketoglutarate (CAS. 13192–04–6) was from Cayman.

### Western blot analysis

Western blot analyses were performed as described (19). Briefly, the cells were collected following different treatment conditions and lysed with lysis buffer. Protein concentrations were determined by Bradford assay (BIO-RAD), and lysate proteins in the loading buffer were denatured by boiling for 5 min. Lysate proteins were resolved on Tris-glycine protein gels (Life Technologies) and then transferred to PVDF membranes (PerkinElmer). Membranes were blocked with milk (5%) or BSA (10%) in TBST for at least 1 h and incubated with primary antibody dilutions in TBST overnight at 4 °C. Horseradish-peroxidase-conjugated secondary antibodies were applied to detect primary antibodies, followed by imaging with Western Lighting Plus-ECL (PerkinElmer).

The following antibodies were used for Western blot. The GLS antibody was raised against the sequence-KLDPRREGGDQRHS and GLS2 antibody was obtained from ProSci (Cat. 6217). Phospho-c-Jun (Ser73) antibody (Cat. 9164), Phospho-c-Jun (Ser63) antibody (Cat. 9261), c-Jun (60A8) rabbit mAb antibody (Cat. 9165), vinculin (E1E9V) XP rabbit mAb antibody (Cat. 13901), anti-rabbit IgG, HRP-linked antibody (Cat. 7074), anti-mouse IgG, and HRP-linked antibody (cat. 7076) were all obtained from Cell Signaling Technology. Anti-coronavirus antibody, OC43 strain, clone 541-8F (Cat. MAB9012) was from Millipore Sigma. SARS-CoV-2/2019-nCoV Spike/S2 Antibody (Cat. 40,590-T62), and HCoV-229E Nucleocapsid Antibody (Cat. 40,640-T62) were from Sino Biological. Alkaline phosphatase-conjugated donley anti-mouse IgG (H  + L) was from Jackson Immunoresearch (Cat. 715–056–150). NBT/BCIP substrate was from Thermofisher (Cat. WP20001).

### Immunofluorescence staining

HBECs were grown in the 4-well slide chamber and were infected with diluted HCoV-OC43 viruses (MOI 0.01) for 1 h at 33 °C, 5%CO_2_. The culture medium was changed to growth medium, and the cells were maintained at 37 °C, 5%CO_2_, for 22 h. One drop of NucBlue Live Cell staining solution (Invitrogen, Cat. R37605) was added to each cell well for 1 h. The cells were fixed with 3.7% formaldehyde for 20 min and washed with PBS (3X). GLS antibody (1:500, the same antibody used at Western blot) was added to the cell chamber at room temperature for 2 h, the cells were washed with PBS (3X), and then HCoV-OC43 antibody (1:500, the same antibody as Western blot) was added to the chamber for 2 h at room temperature. The mixture of goat anti-rabbit secondary antibody Alexa Fluor 568 (1:200) (Thermo Fisher, Cat. A-11036) for GLS primary antibody, and goat anti-mouse secondary antibody Alexa Fluor 488 (1:200) (Thermo Fisher, Cat. A-11001) for HCoV-OC43 primary antibody, were applied to the chamber and incubated at room temperature for 1 h with rocking. The cells were washed with PBS (3X). The images were taken with a KEYENCE BZ-X810 microscope.

### RNA isolation from cells, media, and mouse tissue, and quantitative PCR (qPCR)

All SARS-CoV-2 related studies, including cells and mice, were conducted in a Biosafety Level 3 (BSL3) facility at Cornell University. The SARS-CoV-2-containing materials were heat-inactivated, and TRIzol solution was added before RNA isolation. Total RNA was isolated from cells, tissues, or media using a Direct-zol RNA MicroPrep kit (ZYMO Research, cat. R2062) or a NucleoSpin RNA Virus kit (Takara, cat. 740956), respectively. qPCR analysis was carried out with specific primers, and iTaq Universal SYBR Green One-step kit (BIO-RAD, cat.1725151) for total RNA from cells and media of HCoV-OC43 virus infection, and iTaq Universal Probes one-step kit (BIO-RAD, cat.1725141) for SARS-CoV-2 related materials. Reactions were performed using the real-time PCR system (ViiA7 applied biosystems).

The primers were used for this study:

SARS-CoV-2-spike-F (TGGCCGCAAATTGCACAATT)

SARS-CoV-2-spike-R (TGTAGGTCAACCACGTTCCC)

SARS-COV-2 probe (FAM/CGCATTGGCATGGAAGTCAC/BHQ)

HCoV-OC43-F (CCCAAGCAAACTGCTACCTCTCAG)

HCoV-OC43-R (CCCAAGCAAACTGCTACCTCTCAG)

HCoV-229E-F (TCTGCCAAGAGTCTTGCTCG)

HCoV-229E-R (TCTGCCAAGAGTCTTGCTCG)

GLS-F (TGTCACGATCTTGTTTCTCTGTG)

GLS-R (TCATAGTCCAATGGTCCAAAG)

GLS2-F (GCCTGGGTGATTTGCTCTTTT)

GLS2-R (CCTTTAGTGCAGTGGTGAACTT)

actin-F (CATCGAGCACGGCATCGTCA)

actin-R (TAGCACAGCCTGGATAGCAAC)

### Metabolite extractions

HBECs were grown in 6-well plates at 80% confluence (triplets for each condition), pretreated with SU1 (2.5μM) or UP4 (1μM) for 3 h with non-treated wells as controls, and then infected with HCoV-OC43 (MOI 0.01) for 1 h at 33 °C, 5%CO_2_, with shaking, followed by the media being changed to growth media with or without inhibitors, and the incubations then continued at 37 °C, 5%CO_2_, for 23 h. The cell plates were placed on dry ice, and 1 ml of ice-cold extraction solution (containing 50% methanol, 30% acetonitrile and 20% H_2_O) was added to each well of the cells with the cells remaining on dry ice for 10 min. The cells were scraped in the extraction solution and transferred to Eppendorf tubes. The tubes were incubated on dry ice for 1 h and then centrifuged at 13,000 rpm for 10 min. Supernatants (700μl) from each sample were transferred to new tubes, and then samples were analyzed at the Cold Spring Harbor Metabolomic Facility.

### Metabolomics analysis

Metabolite levels were determined for targeted metabolomics analysis of HBECs that were either uninfected, HCoV-OC43-infected, or HCoV-OC43-infected and treated with UP4 or SU1. The heatmaps of different metabolic pathways were produced using the Cluster Analysis module under the same platform using the metabolite levels in individual pathways as inputs with the same parameters.

### Genetic knockdowns using shRNA and siRNA

Knockdowns of GLS expression in HBECs cells were achieved using short hairpin RNA (shRNA). Lentivirus particles for each shRNA construct were generated using exponentially growing 293T cells (ATCC) as described previously (11, 28). Silencer Select pre-designed siRNAs targeting GLS and the control silencer were transfected into HBECs, using 60 mm dishes, with 0.3 ml Opti-MEM(GIBCO) containing 100nM of the appropriate siRNA to give the final concentration of 10nM, along with 0.3 ml Opti-MEM containing 12 μl of Lipofectamine 2000 (Invitrogen), were incubated separately at room temperature for 5 min. The two solutions were then combined and incubated for an additional 20 min, mixed with 2.4 ml culture medium, and added to cells. After 5 h of incubation at 37 °C, the transfection mixture was replaced with fresh culture medium. For all knockdowns, two independent shRNAs or siRNAs were used, along with negative control shRNA or siRNA.

The following shRNA and siRNA constructs pLKO.1-puro shRNA control plasmid (Cat. SHC002), pLKO.1-TRCN0000051135 targeting GLS (Clone ID: NM_014905.2–1441s1c1), and pLKO.1-TRCN0000298987 targeting GLS (Clone ID: 014,905.3–1475s21c1), were from Millipore Sigma. Silencer Select Negative Control No.1 siRNA (Cat. 4390843), Silencer Select GLS siRNA (ID. s5838), and Silencer Select GLS2 siRNA (ID. 5840), were from Thermo Fisher Scientific.

### Virus plaque assay

A total of 6 × 10^5^ host cells were seeded in 12-well plates or 1 × 10^6^ cells in 6-well plates with culture medium and incubated for 24 h at 37 °C. The wells were washed with PBS and then infected with mock supernatant or different dilutions of virus (200–500 μl). The viral samples are either from the virus stock, from the virus infected cell lysates with different conditions, or from the media of virus infected cells with different conditions, in triplicate per condition. The plates were incubated at 37 °C for 1 h on a rocker. Sterile 0.4% Oxoid agar in culture medium was prepared and 1 ml (12-well plate) or 2 ml (6-well plate) was applied to each well to replace the medium. The cell plates were placed in a tissue culture hood for 15 min as the agar overlay turned solid, then incubated at 37 °C, 5%CO_2_, for 5 to 7 days. PFA (4%) was used to fix the cells for 30 min at room temperature, and then the agar was removed. Following immunostaining with virus antibody the plaques were counted as described above. In another method we used, PFA fixed cells were washed with PBS and permeabilized with 0.2% Triton X-100 in PBS for 10 min, followed by PBS washing. HCoV-OC43 viruses were stained with anti-OC43 antibody (Millipore, MBA9012) in 1% BSA (1:500 dilution) for 2 h at room temperature. After washing with PBS three times, alkaline phosphatase-conjugated donkey anti-mouse IgG (H  + L) secondary antibody (Jackson Immunoresearch, 715–056–150) for 1 h at room temperature on a rocker. The cells were washed with PBS, 500 μl of 1-Step NBT/BCIP substrate was applied, followed by incubating in the dark for 30 min. The reaction was stopped by washing with PBS three times. The plates were allowed to dry and plaques were counted.

### SARS-CoV-2 infection and treatment in mice

Studies in mice were performed following the recommendations in the Guide for the Care and Use of Laboratory Animals of the National Institutes of Health. All protocols were performed under BSL3 conditions and approved by The CARE of Cornell University (protocol number 2016–0097). Eight-week-old heterozygous K18-hACE2 c57BL/6J mice (strain 2B6.Cg-Tg(K18-hACE2)2Prlmn/J) were used for this study (kindly provided by the lab of Avery August of Cornell University, originally from The Jackson Laboratory). Mice were intranasally inoculated with 2 × 10^3^ PFU per mouse using passage 1 of a single-plaque isolated virus propagated from USA-WA1/2020 (BEI resources; NR-52281). Daily 100 μl mixtures of 5% DMSO with or without inhibitors, 5% ethanol, and 5% Cremophor EL were administered through intraperitoneal injection (IP).

### Statistical analyses

All data were analyzed with one-way ANOVA with Bonferroni correction and each experiment was repeated independently at least three times.

## Data availability

Metabolomics data is available on request.

## Supporting information

This article contains supporting figures.

## Conflict of interest

The authors declare that they have no conflicts of interest with the contents of this article.

## References

[1] Kesheh M.M., Hosseini P., Soltani S., Zandi M. (2022). An overview on the seven pathogenic human coronaviruses. Rev. Med. Virol..

[2] Tang G., Liu Z., Chen D. (2022). Human coronaviruses: origin, host and receptor. J. Clin. Virol..

[3] V’kovski P., Kratzel A., Steiner S., Stalder H., Thiel V. (2021). Coronavirus biology and replication: implications for SARS-CoV-2. Nat. Rev. Microbiol..

[4] Zhou P., Yang X.L., Wang X.G., Hu B., Zhang L., Zhang W. (2020). A pneumonia outbreak associated with a new coronavirus of probable bat origin. Nature.

[5] Beyerstedt S., Casaro E.B., Rangel É.B. (2021). COVID-19: angiotensin-converting enzyme 2 (ACE2) expression and tissue susceptibility to SARS-CoV-2 infection. Eur. J. Clin. Microbiol. Infect. Dis..

[6] Shapira T., Monreal I.A., Dion S.P., Buchholz D.W., Imbiakha B., Olmstead A.D. (2022). A TMPRSS2 inhibitor acts as a pan-SARS-CoV-2 prophylactic and therapeutic. Nature.

[7] Liu D.X., Liang J.Q., Fung T.S. (2021). Encyclopedia of Virology.

[8] Sriwilaijaroen N., Suzuki Y. (2020). Host receptors of influenza viruses and coronaviruses—molecular mechanisms of recognition. Vaccines.

[9] Tang A.T., Buchholz D.W., Szigety K.M., Imbiakha B., Gao S., Frankfurter M. (2023). Cell-autonomous requirement for ACE2 across organs in lethal mouse SARS-CoV-2 infection. PLoS Biol..

[10] Hulswit R.J.G., Lang Y., Bakkers M.J.G., Li W., Li Z., Schouten A. (2019). Human coronaviruses OC43 and HKU1 bind to 9-O-acetylated sialic acids via a conserved receptor-binding site in spike protein domain A. Proc. Natl. Acad. Sci..

[11] Li Z., Tomlinson A.C., Wong A.H., Zhou D., Desforges M., Talbot P.J. (2019). The human coronavirus HCoV-229E S-protein structure and receptor binding. eLife.

[12] Chambers J.W., Maguire T.G., Alwine J.C. (2010). Glutamine metabolism is essential for human cytomegalovirus infection. J. Virol..

[13] Mayer K.A., Stöckl J., Zlabinger G.J., Gualdoni G.A. (2019). Hijacking the supplies: metabolism as a novel facet of virus-host interaction. Front. Immunol..

[14] Fontaine K.A., Camarda R., Lagunoff M. (2014). Vaccinia virus requires glutamine but not glucose for efficient replication. J. Virol..

[15] Finley L.W.S. (2023). What is cancer metabolism?. Cell.

[16] de la Rosa V., Campos-Sandoval J.A., Martín-Rufián M., Cardona C., Matés J.M., Segura J.A. (2009). A novel glutaminase isoform in mammalian tissues. Neurochem. Int..

[17] Pavlova N.N., Thompson C.B. (2016). The emerging hallmarks of cancer metabolism. Cell Metab..

[18] De Berardinis RJ., Chandel N.S. (2016). Fundamentals of cancer metabolism. Sci. Adv..

[19] Greene K.S., Lukey M.J., Wang X., Blank B., Druso J.E., Lin M.C.J. (2019). SIRT5 stabilizes mitochondrial glutaminase and supports breast cancer tumorigenesis. Proc. Natl. Acad. Sci. U. S. A..

[20] Vander Heiden M.G., DeBerardinis R.J. (2017). Understanding the intersections between metabolism and cancer biology. Cell.

[21] Sanchez E.L., Pulliam T.H., Dimaio T.A., Thalhofer A.B., Delgado T., Lagunoff M. (2017). Glycolysis, glutaminolysis, and fatty acid synthesis are required for distinct stages of kaposi’s sarcoma-associated herpesvirus lytic replication. J. Virol..

[22] DeBerardinis R.J., Lum J.J., Hatzivassiliou G., Thompson C.B. (2008). The biology of cancer: metabolic reprogramming fuels cell growth and proliferation. Cell Metab..

[23] Altman B.J., Stine Z.E., Dang C.V. (2017). From krebs to clinic : glutamine metabolism to cancer therapy. Nat. Rev. Cancer.

[24] Ferrer C.M., Lynch T.P., Sodi V.L., Falcone J.N., Schwab L.P., Peacock D.L. (2014). O-GlcNAcylation regulates cancer metabolism and survival stress signaling via regulation of the HIF-1 pathway. Mol. Cell.

[25] Katt W.P., Lukey M.J., Cerione R.A. (2017). A tale of two glutaminases: homologous enzymes with distinct roles in tumorigenesis. Future Med. Chem..

[26] Campos-Sandoval J.A., Martín-Rufián M., Cardona C., Lobo C., Peñalver A., Márquez J. (2015). Glutaminases in brain: multiple isoforms for many purposes. Neurochem. Int..

[27] Wang J Bin, Erickson J.W., Fuji R., Ramachandran S., Gao P., Dinavahi R. (2010). Targeting mitochondrial glutaminase activity inhibits oncogenic transformation. Cancer Cell.

[28] Lukey M.J., Cluntun A.A., Katt W.P., Lin M.C.J., Druso J.E., Ramachandran S. (2019). Liver-type glutaminase GLS2 is a druggable metabolic node in luminal-subtype breast cancer. Cell Rep..

[29] Gao P., Tchernyshyov I., Chang T.C., Lee Y.S., Kita K., Ochi T. (2009). C-Myc suppression of miR-23a/b enhances mitochondrial glutaminase expression and glutamine metabolism. Nature.

[30] Lukey M.J., Greene K.S., Erickson J.W., Wilson K.F., Cerione R.A. (2016). The oncogenic transcription factor c-Jun regulates glutaminase expression and sensitizes cells to glutaminase-targeted therapy. Nat. Commun..

[31] Dias M.M., Adamoski D., dos Reis L.M., Ascenção C.F.R., de Oliveira K.R.S., Mafra A.C.P. (2020). GLS2 is protumorigenic in breast cancers. Oncogene.

[32] Milano S.K., Huang Q., Nguyen T.T.T., Ramachandran S., Finke A., Kriksunov I. (2022). New insights into the molecular mechanisms of glutaminase C inhibitors in cancer cells using serial room temperature crystallography. J. Biol. Chem..

[33] Wicker C.A., Hunt B.G., Krishnan S., Aziz K., Parajuli S., Palackdharry S. (2021). Glutaminase inhibition with telaglenastat (CB-839) improves treatment response in combination with ionizing radiation in head and neck squamous cell carcinoma models. Cancer Lett..

[34] Gross M.I., Demo S.D., Dennison J.B., Chen L., Chernov-Rogan T., Goyal B. (2014). Antitumor activity of the glutaminase inhibitor CB-839 in triple-negative breast cancer. Mol. Cancer Ther..

[35] de Oliveira L.G., de Souza Angelo Y., Yamamoto P., Carregari V.C., Crunfli F., Reis-de-Oliveira G. (2022). SARS-CoV -2 infection impacts carbon metabolism and depends on glutamine for replication in Syrian hamster astrocytes. J. Neurochem..

[36] Bharadwaj S., Singh M., Kirtipal N., Kang S.G. (2021). SARS-CoV-2 and glutamine: SARS-CoV-2 triggered pathogenesis via metabolic reprograming of glutamine in host cells. Front. Mol. Biosciences.

[37] Thaker S.K., Ch’ng J., Christofk H.R. (2019). Viral hijacking of cellular metabolism. BMC Biol..

[38] Thai M., Thaker S.K., Feng J., Du Y., Hu H., Ting Wu T. (2015). MYC-induced reprogramming of glutamine catabolism supports optimal virus replication. Nat. Commun..

[39] Schultz D.C., Johnson R.M., Ayyanathan K., Miller J., Whig K., Kamalia B. (2022). Pyrimidine inhibitors synergize with nucleoside analogues to block SARS-CoV-2. Nature.

[40] Chen J., Ye C., Wan C., Li G., Peng L., Peng Y., Fang R. (2021). The roles of c-jun N-terminal kinase (JNK) in infectious diseases. Int. J. Mol. Sci..

[41] Mizutani T., Fukushi S., Saijo M., Kurane I., Morikawa S. (2005). JNK and PI3k/Akt signaling pathways are required for establishing persistent SARS-CoV infection in Vero E6 cells. Biochim. Biophys. Acta (BBA) - Mol. Basis Dis..

[42] Varshney B., Lal S.K. (2011). SARS-CoV accessory protein 3b induces AP-1 transcriptional activity through activation of JNK and ERK pathways. Biochemistry.

[43] Leite F.G.G., Torres A.A., De Oliveira L.C., Da Cruz A.F.P., Soares-Martins J.A.P., Pereira A.C.T.C. (2017). c-Jun integrates signals from both MEK/ERK and MKK/JNK pathways upon vaccinia virus infection. Arch. Virol..

[44] McDermott L.A., Iyer P., Vernetti L., Rimer S., Sun J., Boby M. (2016). Design and evaluation of novel glutaminase inhibitors. Bioorg. Med. Chem..

[45] Katt W.P., Ramachandran S., Erickson J.W., Cerione R.A. (2012). Dibenzophenanthridines as inhibitors of glutaminase C and cancer cell proliferation. Mol. Cancer Ther..

[46] Stalnecker C a, Ulrich S.M., Li Y., Ramachandran S., McBrayer M.K., DeBerardinis R.J. (2014). Mechanism by which a recently discovered allosteric inhibitor blocks glutamine metabolism in transformed cells. Proc. Natl. Acad. Sci. U. S. A..

[47] Robinson M.M., Mcbryant S.J., Tsukamoto T., Rojas C., Ferraris D.V., Hamilton S.K. (2007). Novel mechanism of inhibition of rat kidney-type glutaminase by bis-2-(5-phenylacetamido-1,2,4-thiadiazol-2-yl)ethyl sulfide (BPTES). Biochem. J..

[48] Huang Q., Stalnecker C., Zhang C., McDermott L.A., Iyer P., O'Neill J. (2018). Characterization of the interactions of potent allosteric inhibitors with glutaminase C, a key enzyme in cancer cell glutamine metabolism. J. Biol. Chem..

[49] McCray P.B., Pewe L., Wohlford-Lenane C., Hickey M., Manzel L., Shi L. (2007). Lethal infection of K18- *hACE2* mice infected with severe acute respiratory syndrome coronavirus. J. Virol..

[50] Winkler E.S., Bailey A.L., Kafai N.M., Nair S., McCune B.T., Yu J. (2020). SARS-CoV-2 infection of human ACE2-transgenic mice causes severe lung inflammation and impaired function. Nat. Immunol..

[51] Johansen M.D., Irving A., Montagutelli X., Tate M.D., Rudloff I., Nold M.F. (2020). Animal and translational models of SARS-CoV-2 infection and COVID-19. Mucosal Immunol..

[52] Best S.A., Gubser P.M., Sethumadhavan S., Kersbergen A., Negrón Abril Y.L., Goldford J. (2022). Glutaminase inhibition impairs CD8 T cell activation in STK11-/Lkb1-deficient lung cancer. Cell Metab..

[53] Jiang B., Zhang J., Zhao G., Liu M., Hu J., Lin F. (2022). Filamentous GLS1 promotes ROS-induced apoptosis upon glutamine deprivation via insufficient asparagine synthesis. Mol. Cell.

[54] Feng S., Aplin C., Nguyen T.T.T., Milano S.K., Cerione R.A. (2024). Filament formation drives catalysis by glutaminase enzymes important in cancer progression. Nat. Commun..

[55] McDermott L., Koes D., Mohammed S., Iyer P., Boby M., Balasubramanian V. (2019). GAC inhibitors with a 4-hydroxypiperidine spacer: requirements for potency. Bioorg. Med. Chem. Lett..

[56] Harding J.J., Telli M., Munster P., Voss M.H., Infante J.R., DeMichele A. (2021). A phase I dose-escalation and expansion study of telaglenastat in patients with advanced or metastatic solid tumors. Clin. Cancer Res..

[57] Riess J.W., Frankel P., Shackelford D., Dunphy M., Badawi R.D., Nardo L. (2021). Phase 1 trial of MLN0128 (Sapanisertib) and CB-839 HCl (telaglenastat) in patients with advanced NSCLC (NCI 10327): rationale and study design. Clin. Lung Cancer.

[58] Seifert M., Bera S.C., Van Nies P., Kirchdoerfer R.N., Shannon A., Le T.T.N. (2021). Inhibition of sars-cov-2 polymerase by nucleotide analogs from a single-molecule perspective. eLife.

[59] Mouffouk C., Mouffouk S., Mouffouk S., Hambaba L., Haba H. (2021). Flavonols as potential antiviral drugs targeting SARS-CoV-2 proteases (3CLpro and PLpro), spike protein, RNA-dependent RNA polymerase (RdRp) and angiotensin-converting enzyme II receptor (ACE2). Eur. J. Pharmacol..

[60] Tian L., Qiang T., Liang C., Ren X., Jia M., Zhang J. (2021). RNA-dependent RNA polymerase (RdRp) inhibitors: the current landscape and repurposing for the COVID-19 pandemic. Eur. J. Med. Chem..

[61] Peng F., Yuan H., Wu S., Zhou Y. (2021). Recent advances on drugs and vaccines for COVID-19. Inquiry.

[62] Bai Z., Cao Y., Liu W., Li J. (2021). The SARS-CoV-2 nucleocapsid protein and its role in viral structure, biological functions, and a potential target for drug or vaccine mitigation. Viruses.

[63] Bharadwaj S., Singh M., Kirtipal N., Kang S.G. (2021). SARS-CoV-2 and glutamine: SARS-CoV-2 triggered pathogenesis via metabolic reprograming of glutamine in host cells. Front. Mol. Biosciences.

[64] Ferreira A.P.S., Cassago A., de Gonçalves K.A., Dias M.M., Adamoski D., Ascenção C.F.R. (2013). Active glutaminase C self-assembles into a supratetrameric oligomer that can Be disrupted by an allosteric inhibitor. J. Biol. Chem..

[65] Møller M., Nielsen S.S., Ramachandran S., Li Y., Li Y., Tria G. (2013). Small angle X-ray scattering studies of mitochondrial glutaminase C reveal extended flexible regions, and link oligomeric state with enzyme activity. PLoS One.

