# Supplementary material for: Glutamine metabolism is essential for coronavirus replication in host cells and in mice
Source: J Biol Chem. 2024 Dec 9;301(1):108063. doi: 10.1016/j.jbc.2024.108063 (PMC11750454; doi:10.1016/j.jbc.2024.108063)
Supplement: Supporting Figure Legends [file mmc1.docx]

**Supporting Figures Legends**

**Supporting Figure 1**. **Metabolomic data—Purine axis.** Bar graphs from metabolomics show the metabolite changes in the purine synthetic pathway in HBECs infected with HCoV-OC43 compared to uninfected samples. Data are presented as mean +/-SD (n=3 independent biological replicates).

**Supporting Figure 2. Metabolomic data—Pyrimidine axis.** Bar graphs from metabolomics show the metabolite changes in the pyrimidine synthetic pathway in HBECs infected with HCoV-OC43 compared to uninfected samples. Data are presented as mean +/-SD (n=3 independent biological replicates).

**Supporting Figure 3. Glutaminase inhibitors do not block coronavirus entry.** (A) HBECs were pre-treated with DMSO, 968(2.5mM), BPTES(2.5mM), SU1(1mM), and UP4(0.1mM) for 3 hours, and then the cells were infected with HCoV-OC43 (MOI 0.01) for 1 hour at 33^o^C, 5% CO_2_, and collected. Western blot analysis showing the HCoV-OC43 levels in HBECs in the presence or absence of inhibitors. (B). HBECs were treated with or without inhibitors and infected with virus (or not infected) as above. The viral RNA was isolated from the non-infected or infected cells and analyzed by qPCR. One-way ANOVA with Bonferroni correction was used to determine significance in B, ns indicates the data is not significant.

**Supporting Figure 4. Dose-dependent inhibition of virus-infected HBECs and HCT8 cells by SU1 and UP4.** (A) HBECs were pretreated with different doses of SU1 (0, 0.5, 1, 2.5, 5, 10 µM) for 3 hours, followed by infection with HCoV-OC43 (MOI 0.01, 2%HS RPMI, 33^o^C, 5%CO_2,_ for 1 hour). The cells were changed to the growth media with different concentrations of SU1 and incubated for 23 hours at 37^o^C, 5%CO_2_. Western blot analysis showed the effects of SU1 on HCoV-OC43 replication in HBECs. (B) A similar experiment was performed to examine the effects of the UP4 compound. Western blot analysis showed the effects of varying doses of UP4 (0, 0.1, 0.5, 1, 5, 10 µM) upon a 24-hour virus infection of HBECs. (C) HCT8 cells were pretreated with different concentrations of SU1 (0, 0.1, 0.5, 1, 2.5, 5, 10 µM) for 3 hours and infected with HCoV-OC43, as described above. Western blot analysis shows the different virus levels as a function of SU1 concentrations. (D) HCT8 cells were pretreated with varying doses of UP4 (0, 0.1, 0.5, 1, 5, 10 µM) and then infected with HCoV-OC43. Western blot analysis shows the HCoV-OC43 replication levels as a function of different amounts of UP4.

**Supporting Figure 5. Plaque assays in HBECs and media with different conditions.** HBECs (1X10^6^) were seeded in 6-well plates, pretreated with DMSO, SU1(2.5µM), and UP4(0.1µM) for 3 hours, infected with HCoV-OC43 (MOI 0.01) at 33^o^C, 5%CO_2_, with shaking for 1 hour, at which point the agar medium replaced the virus solution after a PBS wash**.** The plates were maintained at room temperature for 15 minutes and then incubated at 37^o^C, 5%CO_2_, for 4 days. The agar layer was removed, and cells were fixed with 4% PFA and stained with HCoV-OC43 antibody and goat anti-mouse secondary antibody Alexa Fluor 488. Triplicate determinations were performed for each condition. (A) Imaging of the plaques. (B) The bar graph shows the relative plaque numbers for virus-infected cells treated with DMSO ( as control), SU1 or UP4. One-way ANOVA with Bonferroni correction was used to determine significance in B, **** indicates modified *P* < 0.0001. (C) HBECs were infected with HCoV-OC43 and treated with DMSO, 968, SU1 and UP4 for 24 hours as described in the experimental procedures, the media were collected, followed by plaque assay. The data shows the HCoV-OC43 viral titers of each condition of medium. (D) Plaque assay showing HCoV-OC43 viral titers in the viral infected HBEC media expressing a control shRNA or two independent GLS-targeted shRNAs. (E) Plaque assay showing the HCoV-OC43 viral titers in the media of HBECs infected with HCoV-OC43 and treated with UP4 or UP4 and Dm⍺KG.

**Supporting Figure 6. Coronavirus infection affects nucleotide synthesis in host cells.** Heatmap for metabolites in uninfected HBECs, HBECs infected with HCoV-OC43, and infected HBECs treated with SU1(2.5µM) and UP4(1µM) (n=3 for each condition).

**Supporting Figure 7. Effects of UP4 treatment on SARS-CoV-2 infected mice.** (A) qPCR assays of SARS-CoV-2 were performed on total RNA from the brain tissues of mice treated with SU1 (1mg/kg) and control mice (n=4 for each group). (B) Probability of survival of K18-hACE2 mice (n=4 per group) infected with SARS-CoV-2 and treated with vehicle control (blue) or UP4 (1.3 mg/kg, green). (C) Weight change of the two groups shown in (B). (D) qPCR assay of SARS-CoV-2 was performed on total RNA of lung tissues; the analysis was carried out on control (n=4) versus UP4 treated mice (1.3mg/kg, n=2). (E) qPCR assays of SARS-CoV-2 were performed on RNA isolated from the brains of mice treated with UP4 (1.3mg/kg). The analysis was carried out on control (n=2) versus UP4 (1.3mg/kg, n=2). (F) Probability of survival on K18-hACE2 mice (n=4 for control, n=3 for UP4) infected with SARS-CoV-2 and treated with vehicle control (blue) or UP4 (0.65 mg/kg, green). (G) Weight change of K18-hACE2 mice infected with SARS-CoV-2 and treated by intraperitoneal injection with UP4 (0.65mg/kg). (H) qPCR assays of SARS-CoV-2 were performed on total RNA from the lung tissues of mice; the analysis was carried out on control (n=4) versus UP4 treated animals (0.65mg/kg; n=1). One-way ANOVA with Bonferroni correction was used to determine significance in A, D, E and H, **** indicates modified *P* < 0.0001.
